# Aziridination-Based Crosslinking Enables Site-Resolved Elucidation of Lipid Binding and Protein Conformational Changes

**DOI:** 10.1021/jacs.6c08027

**Published:** 2026-10-07

**Authors:** Chia-Lung Tsai, Atiya Tahira Tasnim, Zhenyu Xi, Syuan-Ting Kuo, Gopal Reddy Ramidi, Jiaxin Feng, Michael Ryan Eastabrook, David H. Russell, Xuewu Sui, Xin Yan

**Affiliations:** † Department of Chemistry, 14736 Texas A&M University, 580 Ross Street, College Station, Texas 77843, United States; ‡ Department of Biochemistry and Biophysics, Texas A&M University, 300 Olsen Boulevard, College Station, Texas 77843, United States

## Abstract

Lipid–protein interactions play critical roles in cellular function, yet their site-resolved characterization remains challenging due to the dynamic nature of lipid binding and the limited availability of chemoselective strategies for covalently capturing native lipid–protein contacts. Here, we introduce aziridination-based lipid–protein crosslinking coupled with mass spectrometry (Azi-LPXL-MS), a chemoselective strategy that installs an N–H aziridine moiety onto acyl chains of native unsaturated lipids and enables subsequent crosslinking to proximal lysine residues using the amine-reactive reagent disuccinimidyl suberate (DSS). This integrated workflow simultaneously identifies lipid-binding sites and intraprotein peptide–peptide distance constraints, providing direct insight into lipid binding and binding-induced conformational changes within a single experiment. Application of Azi-LPXL-MS to β-lactoglobulin (BLG) demonstrates these capabilities, consistent with known structural models, while additionally providing solution-phase and conformational information not accessible from static structures alone. The power of the method is further demonstrated in the integral membrane enzyme diacylglycerol acyltransferase 1 (DGAT1). Azi-LPXL-MS reveals phosphatidic acid (PA)-binding sites and maps lipid-dependent conformational rearrangements across both resolved and previously uncharacterized regions. These data further define an allosteric conformational network linking lipid engagement to distal structural changes. Integrated with site-directed mutagenesis and molecular docking, two potential PA-binding sites in DGAT1, located in a surface pocket and an internal chamber, were identified and functionally validated, further supporting the experimental findings. These results establish Azi-LPXL-MS as a site-resolved method for studying lipid–protein interactions, providing insight into how individual lipid species engage proteins and influence their conformations in biological systems.

## Graphical abstract

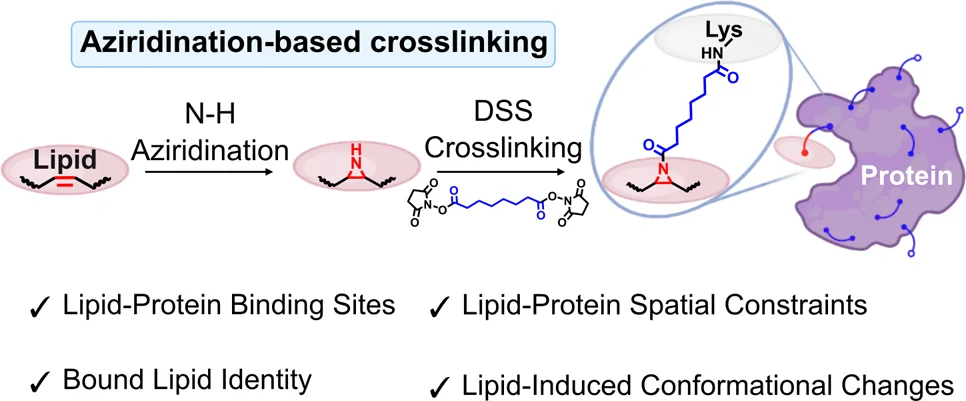

## Introduction

Lipid–protein interactions are fundamental determinants of cellular function, regulating processes such as trafficking, signal transduction, metabolism, and intracellular transport.^1–4^ Disruption of these interactions can compromise membrane integrity, perturb signaling pathways, and impair lipid homeostasis,^5^ contributing to a range of diseases, including neurodegeneration,^6,7^ cardiovascular disorders,^8^ metabolic syndromes,^9^ and cancers.^10,11^ Elucidating the molecular mechanisms underlying lipid–protein interactions is essential for understanding their roles in normal physiology and disease.

Despite their biological importance, the molecular determinants of lipid binding and the associated protein structural consequences remain incompletely understood at the residue level. A key barrier to progress is the chemical nature of lipids. Their structural diversity and limited functional group reactivity hinder the development of strategies capable of capturing interactions with site resolution.^3^ Existing structural approaches provide valuable but incomplete insights. High-resolution structural techniques, such as X-ray crystallography and cryo-electron microscopy (cryo-EM), can define protein architectures but often fail to resolve lipid-binding interfaces because of lipid flexibility and heterogeneity.^12–14^ Biophysical methods such as isothermal titration calorimetry (ITC) and surface plasmon resonance (SPR) provide valuable thermodynamic and kinetic insights; however, ITC often requires large sample quantities, while SPR relies on protein immobilization that can perturb native interactions.^15–18^ Biochemical assays yield qualitative insights without structural detail.^19^ Metabolic labeling captures contacts covalently but may only detect short-range interactions.^20^ These limitations highlight the critical need for complementary approaches to fully understand complex lipid–protein interactions.

Mass spectrometry (MS) has been widely used for investigating biomolecular interactions.^21–31^ Crosslinking mass spectrometry (XL-MS) is a powerful tool for studying protein–protein interactions by covalently linking spatially proximal amino acid residues and identifying them via MS.^32–39^ It provides distance constraints that complement structural and computational methods and can capture dynamic complexes often missed by crystallography or cryo-EM approaches.^40–42^ However, the application of XL-MS to lipid–protein interactions has been limited by the scarcity of chemoselective transformations that can functionalize lipids in a predictable and site-specific manner. Diazirine-based lipids have been developed as photo-crosslinking probes to capture lipid–protein interactions, which can then be analyzed by MS.^43–46^ While these probes are versatile and broadly reactive, their reactivity can also lead to a degree of nonspecific background crosslinking and heterogeneous linkage sites, which may increase the complexity of downstream MS analysis.^47^ In addition, diazirine-based strategies may not efficiently capture protein conformational information during the binding event. Nevertheless, these approaches have established an important foundation and motivate the development of new crosslinking strategies for lipid–protein interaction study.^48^

Here, we develop aziridination-based lipid–protein crosslinking mass spectrometry (Azi-LPXL-MS) to enable site-resolved characterization of lipid–protein interactions (Scheme 1). This strategy leverages aziridination chemistry to install a strained N–H aziridine moiety directly onto lipid acyl chains, creating a well-defined and chemoselective reactive handle while preserving the structural identity of the lipid. Building on our prior work, which established aziridination as an emerging and powerful method for site-specific functionalization of carbon–carbon double (C=C) bonds for lipid structural elucidation at the positional isomer level and for multiplexed quantitative lipidomics,^49–51^ we extend this chemistry to enable covalent capture of lipid–protein interactions. The resulting aziridine functionality facilitates selective crosslinking of lipids to proximal protein residues using crosslinkers such as disuccinimidyl suberate (DSS), capturing lipid–protein complexes with minimal nonspecific reactions. Subsequent MS analysis provides integrated information on lipid species, protein binding sites, and interaction-dependent structural features. Azi-LPXL-MS offers four key capabilities: (1) precise localization of lipid-binding sites, (2) direct spatial constraints between lipids and proteins, (3) structural information on bound lipids, and (4) insights into protein conformational changes upon lipid binding. The method employs a rationally designed crosslinking strategy that selectively targets lipid acyl chains while minimizing interference from lipid head groups, enabling the formation of stable covalent bonds with proximal amino acid residues. This provides a targeted and analytically tractable approach for interrogating lipid–protein interactions at amino acid-level resolution. We demonstrate the method using β-lactoglobulin B (BLG) and the more complex membrane protein, human diacylglycerol acyltransferase 1 (DGAT1), revealing detailed structural features of their lipid interactions.

**Scheme 1:**
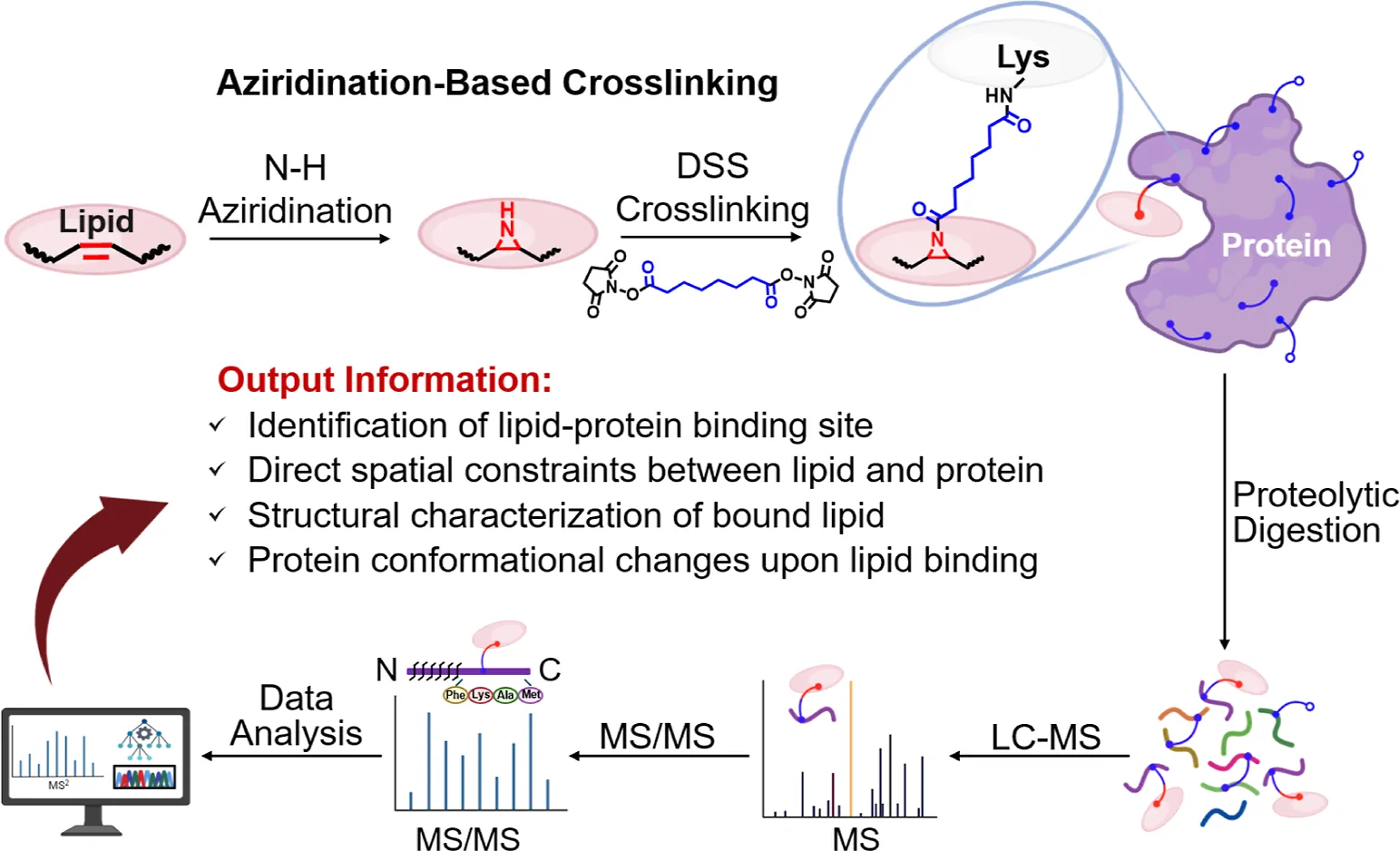
Schematic of the Azi-LPXL-MS Workflow for Site-Resolved Mapping and Structural Characterization of Lipid–Protein Interactions

## Results and Discussion

### Azi-LPXL-MS Enables Site-Resolved Mapping and Structural Characterization of Lipid–Protein Interactions

We first demonstrated the aziridination-enabled lipid derivatization and crosslinking workflow in Azi-LPXL-MS using BLG, a well-characterized lipocalin that binds long-chain fatty acids such as oleic acid (OA) (Figure 1a).^52,53^ OA was converted to aziridinated oleic acid (Azi-OA) via rhodium-catalyzed N–H aziridination using hydroxylamine O-sulfonic acid (HOSA) in hexafluoro-2-propanol (HFIP) (Supporting Information S1). The structure of Azi-OA was confirmed by high-resolution MS (*m*/*z* 298.2726) and MS/MS analysis (Figure S1), which verified efficient installation of the N–H aziridine functionality, as evidenced by characteristic fragment ions at *m*/*z* 142.1587 and 172.1328. We next assessed whether the modified lipid could enable covalent capture of lipid–protein interactions in the presence of a protein crosslinking reagent. Native MS analysis revealed three types of BLG modifications: lipid–protein crosslinks formed between Azi-OA and DSS (Az), DSS-mediated intraprotein crosslinks (D), and hydrolyzed DSS modifications (HD). Incubation of Azi-OA with BLG (18,285 Da) followed by DSS crosslinking yielded lipid–protein crosslinked products (18,720 Da, BLG + Az; 18,858 Da, BLG + Az + D; 18,876 Da, BLG + Az + HD; and 19,014 Da, BLG + Az + HD + D; Figure 1b,c). Notably, we also observed intraprotein crosslinked species (18,423 Da, BLG + D and 18,579 Da, BLG + D + HD; Figure 1b,c). These two product classes capture complementary layers of information: lipid–protein crosslinks directly report on lipid–protein binding events, whereas intraprotein crosslinks reflect protein conformational changes upon lipid binding. These assignments were confirmed by comparing native MS (Figure S2) and deconvoluted mass spectra (Figure 1b) of BLG treated with Azi-OA and DSS against control experiments of apo BLG and BLG treated with DSS alone.

**Figure 1:**
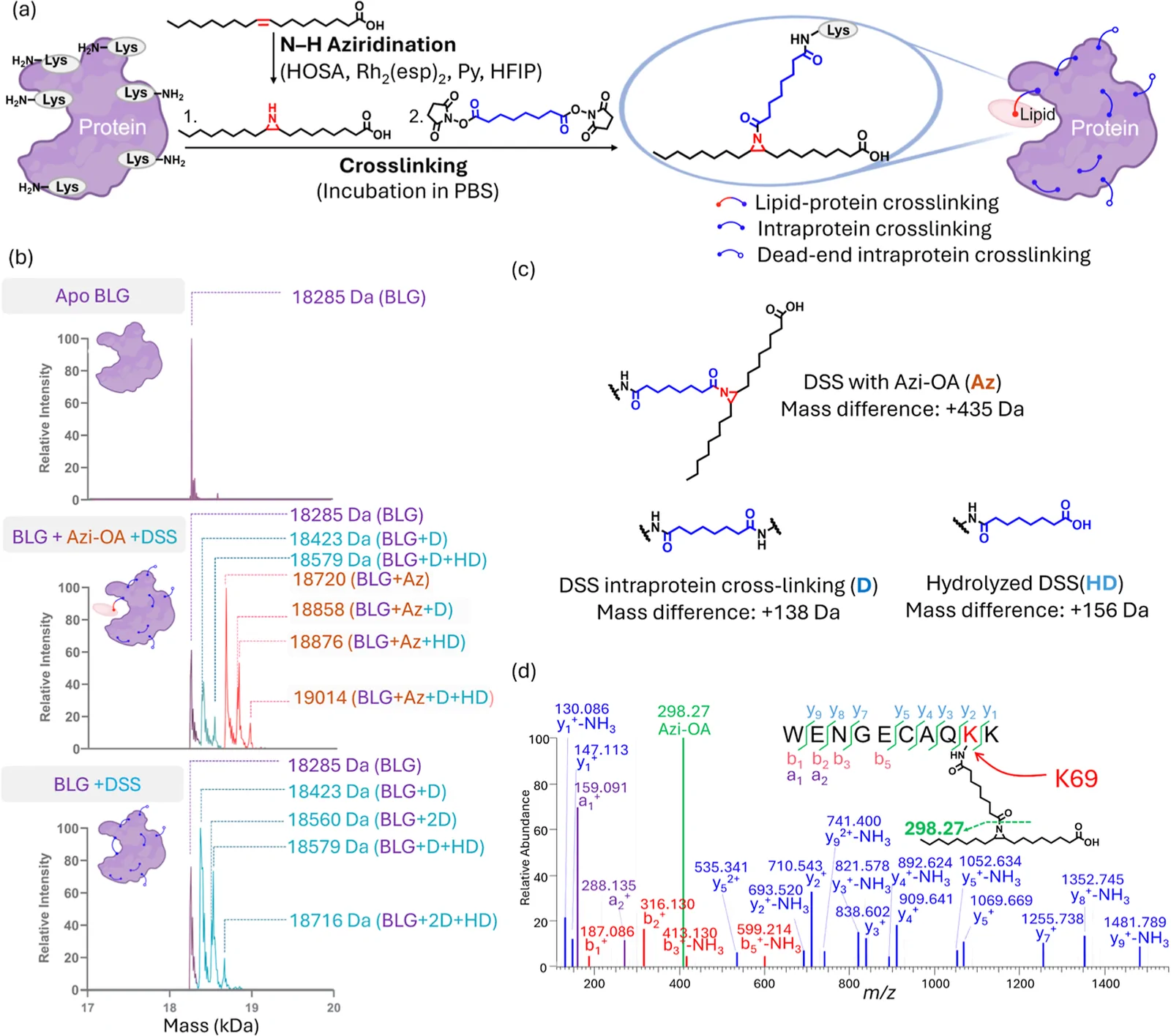
Azi-LPXL-MS study of BLG–lipid interactions. (a) Schematic of the aziridination-enabled crosslinking strategy: OA is converted to Azi-OA, followed by incubation with BLG and subsequent DSS crosslinking prior to XL-MS analysis. (b) Deconvoluted native mass spectra of apo BLG, BLG treated with Azi-OA and DSS, and BLG treated with DSS alone (control), showing mass increases associated with lipid–protein crosslinking and DSS modification. **Az**, lipid–protein crosslink formed between Azi-OA and DSS; **D**, DSS-mediated intraprotein crosslink; and **HD**, hydrolyzed DSS modification. (c) Structures of the crosslinked species observed in (b). (d) HCD MS/MS spectrum of the Azi-OA-modified peptide WENGECAQKK from BLG, demonstrating confident site assignment based on fragment ion coverage.

The equivalents of Azi-OA and DSS were optimized (Supporting Information Section S4) to balance crosslinking efficiency with preservation of downstream MS sequence coverage, as excessive crosslinking can hinder efficient proteolytic digestion, while minimizing nonspecific lipid over-crosslinking.^54^ Based on this optimization, a BLG–Azi-OA–DSS ratio of 1:5:5 was selected for subsequent experiments, yielding crosslinking efficiencies of 58% for lipid–protein products and 21% for intraprotein crosslinking (Supporting Information Section S4). These results show that N–H aziridine-functionalized lipids are compatible with DSS-based crosslinking chemistry and enable simultaneous interrogation of lipid–protein and intraprotein residue interactions within a single workflow.

We next examined whether Azi-LPXL-MS enables precise localization of lipid-binding sites and provides spatial constraints. After protein digestion, LC–MS/MS analysis identified 31 peptide–peptide crosslinks and one lipid–peptide crosslink across three biological replicates (1% FDR) (Figure S5a). The lipid crosslink was unambiguously assigned to K69, supported by a high-confidence MS/MS spectrum (Figure 1d). A mass shift of 563.43 Da between the *y*_1_ and *y*_2_ ions of the peptide WENGECAQKK confirmed K69 as the lipid modification site. Additionally, a characteristic fragment ion at *m*/*z* 298.27, corresponding to Azi-OA, further verified the identity of the bound lipid (Figure 1d).

To validate the location of K69 in the BLG, we further examined the previously reported crystal structure of BLG bound to oleic acid.^52^ The spatial distance between the ε-amino group of K69 and the bound fatty acid in the crystal structure is 11.0 Å (Figure 2d). This distance is consistent with the expected range for DSS-mediated crosslinks (11–20 Å),^55,56^ supporting the feasibility of the observed crosslink and demonstrating that Azi-LPXL-MS provides direct spatial constraints between lipids and proteins and identifies the lipid binding site.

**Figure 2:**
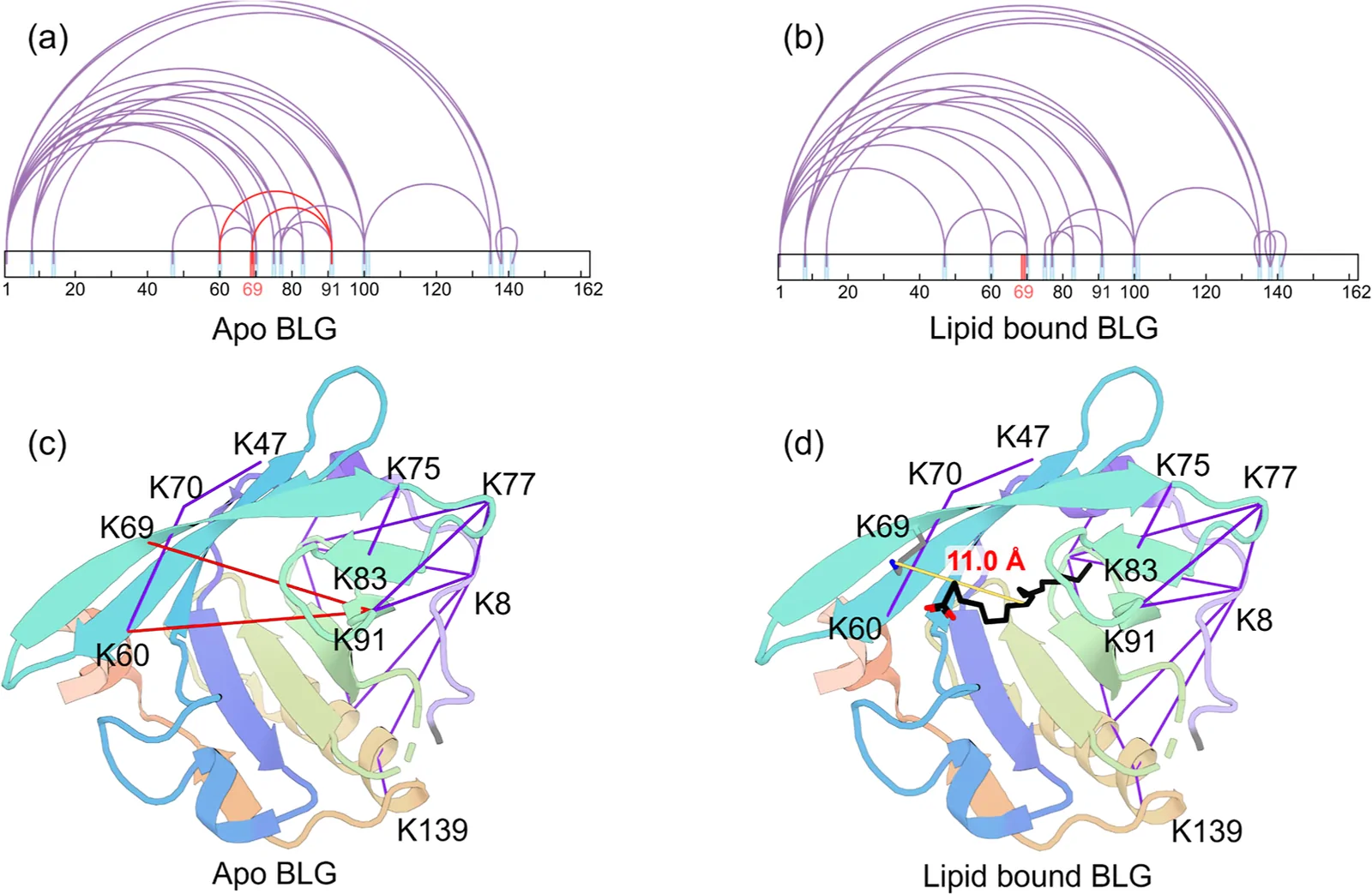
Summary of Azi-LPXL-MS identified lipid–protein and intraprotein crosslinks in apo BLG and lipid-bound BLG. Connecting lines indicate intraprotein crosslinks in (a) apo BLG and (b) lipid-bound BLG. Crosslinks are shown only if they passed 1% FDR filtering and were observed in at least 2 of 3 biological replicates. Notably, no intraprotein crosslinks involving K69 (highlighted in red) were detected in lipid-bound BLG because K69 forms a crosslink with the lipid. Crystal structures of (c) apo BLG and (d) lipid-bound BLG (PDB: 4DQ3) visualize the two crosslinks spanning the entrance of the binding pocket that are present in apo BLG but absent in lipid-bound BLG (red lines). The distance between K69 and the double bond of oleic acid is 11.0 Å (yellow line). The N-terminus (Leu1) of BLG is not resolved in the crystal structure; therefore, the corresponding crosslinks cannot be mapped to the structure (PDB: 4DQ3).

We then investigated whether Azi-LPXL-MS captures lipid-induced conformational dynamics by leveraging its compatibility with intraprotein residue crosslinking. Because the workflow integrates aziridine-mediated lipid–protein crosslinking with DSS-based lysine–lysine crosslinking in a single experiment, changes in protein conformation upon lipid binding can be directly monitored through alterations in intraprotein crosslinking patterns. Crosslinking profiles were highly reproducible across biological replicates. In the apo state, 32 intraprotein crosslinks were identified, and 24 crosslinks were observed in at least 2 of 3 biological replicates, including crosslinks such as K60–K91 and K69–K91 spanning the lipid-binding pocket, supported by high-confidence MS/MS spectra (Figures S6 and S7), and Cα–Cα distance analysis (Supporting Information Section S8). In contrast, these crosslinks were not detected in the lipid-treated BLG. This loss can be attributed to two factors: (1) comparison with the crystal structure (PDB: 4DQ3) indicates that the ε-amino group distances of K60–K91 and K69–K91 increase to 21.8 Å and 22.2 Å, respectively, exceeding the effective span of the DSS crosslinker (11–20 Å), showing lipid-induced conformational change; and (2) lipid binding likely introduces steric occlusion that further restricts access of the crosslinker to these lysine pairs (Figures 2a,b and S10). This loss of crosslinks provides additional structural evidence of ligand engagement and associated conformational information in BLG (Figure 2c,d). Notably, this integrated readout of lipid binding and protein structural change is not readily accessible using conventional photo-crosslinking approaches, which primarily capture proximity without simultaneously reporting on global protein conformational states.^57^

Additionally, Azi-OA crosslinking of denatured BLG was performed to probe the structural dependence of lipid–protein interactions. In contrast to the native protein, the denatured state yielded 5 lipid crosslinking sites and 91 peptide–peptide crosslinks, substantially more than observed for native BLG, consistent with widespread, nonspecific crosslinking driven by increased conformational flexibility and solvent exposure. These results highlight that, whereas denaturation promotes promiscuous lipid attachment, the folded protein constrains reactivity to discrete, site-specific binding interactions (Supporting Information Section S7). All these results demonstrate that Azi-LPXL-MS enables integrated characterization of binding site localization, spatial constraints, lipid identity, and conformational dynamics within a unified experimental framework.

### DGAT1–PA Interaction Revealed by Azi-LPXL-MS

We next applied Azi-LPXL-MS to investigate the interaction between phosphatidic acid (PA) and human DGAT1, an integral membrane enzyme that catalyzes the terminal step of triacylglycerol (TAG) biosynthesis at the endoplasmic reticulum (ER) and functions as a homodimer (∼110 kDa).^58^ DGAT1 occupies a central position in glycerolipid metabolism, and genetic mutations in DGAT1 are directly linked to metabolic disorders.^59^ Although previous studies have resolved the overall structure of DGAT1 and elucidated its catalytic mechanism, where fatty acyl-CoA enters through a cytosolic tunnel and diacylglycerol (DAG) is recruited via a lateral gate to a membrane-embedded active site centered on His415, the molecular details of its lipid interaction landscape remain largely undefined.^60,61^ PA serves as a key metabolic intermediate and an anionic lipid capable of engaging protein surfaces. Previous studies have reported interactions between PA and plant homologs such as *Brassica napus* DGAT1 (BnaDGAT1),^62^ suggesting that such contacts may be conserved. These features make the PA–DGAT1 system a relevant and technically challenging context in which to evaluate our approach for site-resolved mapping of lipid–protein interactions.

To study the interaction between PA and DGAT1, we applied the Azi-LPXL-MS workflow. 1-Palmitoyl-2-oleoyl-*sn*-glycero-3-phosphatidate (POPA) was aziridinated to introduce a chemoselective N–H aziridine handle on the *sn*-2 oleate chain of POPA to form Azi-PA, as confirmed by high-resolution MS (*m*/*z* 690.5073) and tandem MS (Figure S14). Purified human DGAT1 in detergent was then incubated with Azi-PA and DSS to simultaneously capture lipid–protein and intraprotein residue crosslinks within the same experiment. Subsequent LC–MS/MS analysis consistently identified two PA crosslinking sites at K309 and K312 across three biological replicates, supported by a high-confidence HCD MS/MS spectrum and with high reproducibility (Figure 3a). Notably, we also observed a neutral loss corresponding to the PA headgroup with one acyl chain (C_19_H_37_O_6_P) in MS/MS fragmentation of Azi-PA-modified peptides, a characteristic signature that provides additional confirmation of lipid attachment.

**Figure 3:**
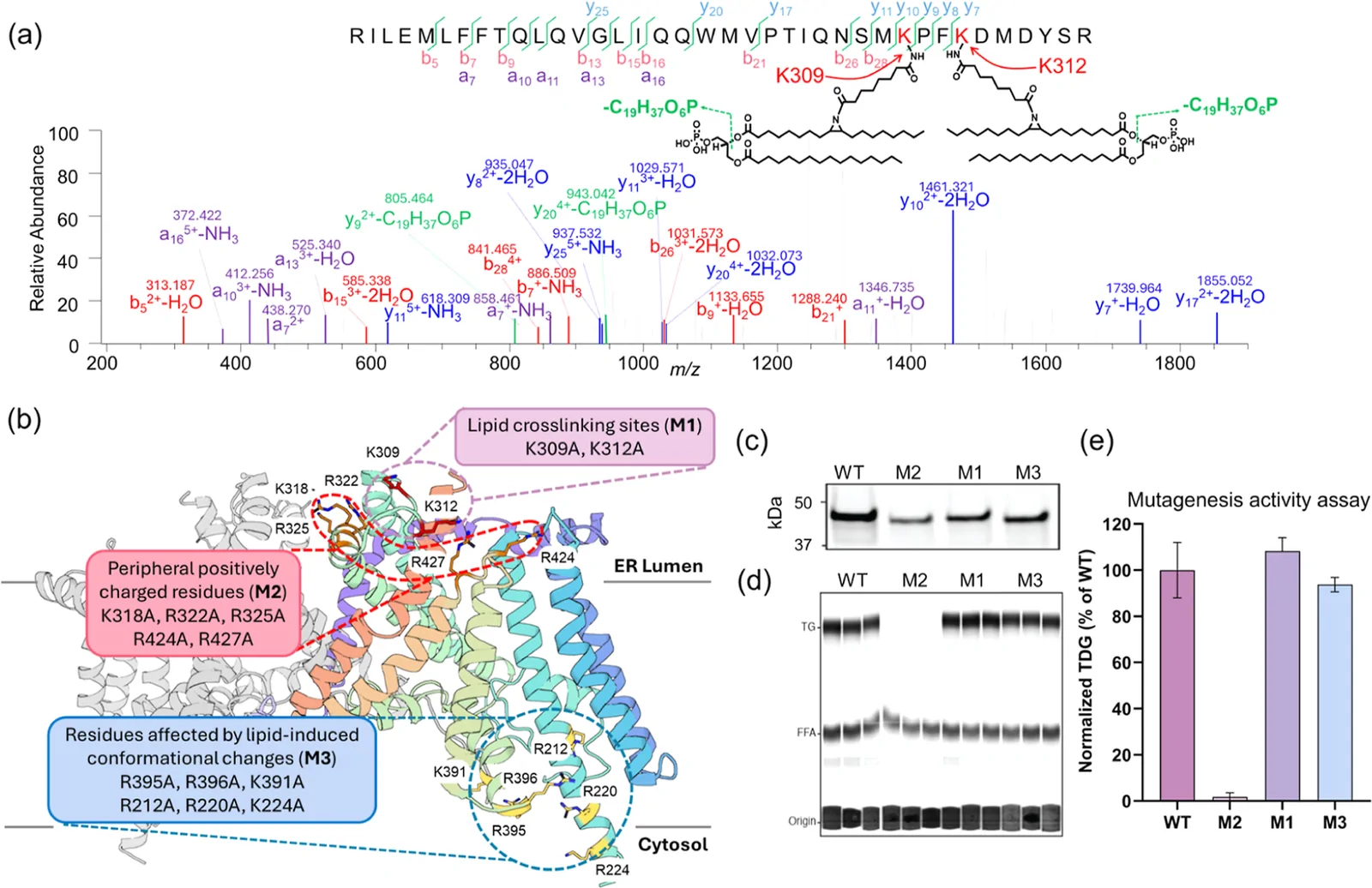
(a) HCD MS/MS spectrum of the PA-crosslinked peptide from human DGAT1, identifying PA binding at K309 and K312. (b) Mutagenesis targeting three classes of residues: (M1) lipid crosslinking sites; (M2) peripheral positively charged residues within 12 Å of the lipid crosslinking sites; and (M3) lipid-induced conformational change sites identified by peptide–peptide crosslinks. (c) Western blot analysis showing that all mutants were expressed at levels comparable to WT DGAT1. (d) TLC analysis of TG formation by WT DGAT1 and mutants, TG: triglycerides and FFA: free fatty acid. (e) Quantification of TG formation shown in (d), normalized to the WT control.

Importantly, intraprotein crosslinks detected from the same experiment revealed two peptide pairs (K224–K391 and K53–K231) that were not observed in apo DGAT1 analysis (Figure S17). This indicates that the observed DSS-mediated peptide–peptide crosslinks arise from the lipid-bound state of DGAT1. Furthermore, the K224–K391 and K53–K231 peptide–peptide crosslinks were also observed in POPA-treated DGAT1, suggesting that their formation is induced by PA binding rather than by the aziridination modification (Supporting Information Section S11). These lipid-dependent crosslinks suggest that PA binding induces protein conformational rearrangements that bring distal lysine residues into closer spatial proximity. Notably, K53 is located within a region unresolved in the cryo-EM structure, highlighting the ability of Azi-LPXL-MS to probe dynamic or structurally ambiguous regions and to provide complementary insights into lipid-induced structural remodeling that are not accessible by conventional structural methods.

To evaluate the functional relevance of our findings, we performed site-directed mutagenesis targeting three classes of residues (Figure 3b): (1) the directly crosslinked lysine residues (M1 mutants: K309A and K312A); (2) neighboring positively charged residues within the length of DSS (∼12 Å) of K309 and K312 (M2 mutants: K318A, R322A, R325A, R424A, and R427A), which may interact with the phosphate headgroup of POPA; and (3) residues identified through changes in intraprotein peptide crosslinking patterns (M3 mutants: R395A, R396A, K391A, KR212A, R220A, and K224A), which likely reflect ligand-induced conformational changes in DGAT1 upon Azi-PA binding, a strategy traditionally used to infer ligand-binding sites.

We expressed wild-type DGAT1 and each mutant fused with a GFP tag in yeast cells, and the expression of each construct was confirmed by Western blot analysis. All mutants were expressed to a level similar to that of the wild-type protein (Figure 3c). While mutation of K309 and K312 to Ala (M1) did not significantly change TAG production, mutation of surrounding positively charged residues (M2) led to a pronounced decrease in TAG production (Figure 3d,e). This result indicates that although K309 and K312 represent direct lipid binding sites, nearby Lys/Arg residues are functionally important for interacting with the PA headgroups.

In contrast, mutation of residues identified from peptide–peptide crosslink changes (M3) exhibited minimal effect on DGAT1 activity. The M3 mutant activity result demonstrates a key limitation of conventional crosslinking-MS methods in studying ligand–protein interaction.^21^ Inferring ligand-binding sites solely from peptide–peptide crosslink changes can be misleading, as protein conformational changes (M3) do not necessarily coincide with direct ligand contact. Azi-LPXL-MS overcomes this limitation by providing identification of the lipid-binding site, distinguishing between structural rearrangements and true ligand engagement (Figure 3e). Notably, the PA-binding site (K309 and K312) is located close to the ER luminal side, whereas the lipid-induced conformational change site (K224–K391) is positioned on the cytosolic side near the fatty acyl-CoA entry region (Figures 3b and 4). This spatial separation suggests that PA binding in the ER lumen allosterically influences DGAT1 structural dynamics at the cytosolic side. These results demonstrate that Azi-LPXL-MS enables detailed characterization of lipid binding sites and lipid-induced conformational changes in DGAT1–PA interactions, facilitating our ongoing mechanistic studies.

**Figure 4:**
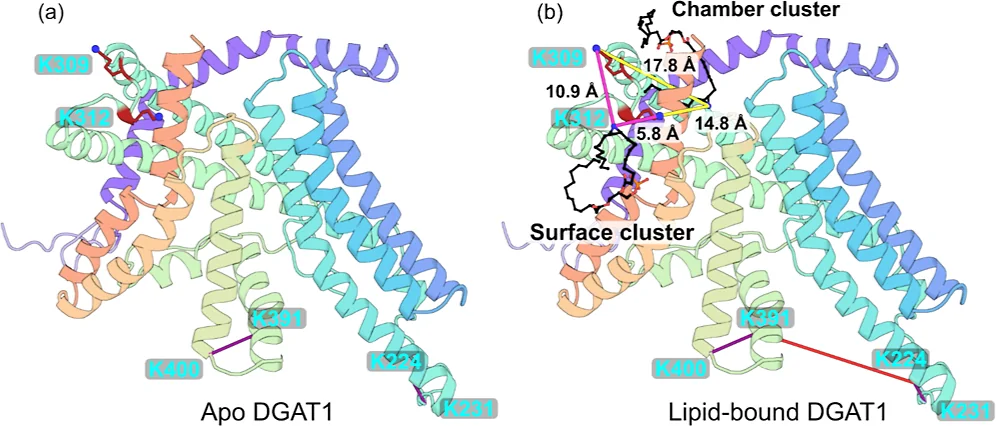
Azi-LPXL-MS-identified lipid–protein and intraprotein crosslinks in apo DGAT1 and lipid-bound DGAT1. Crosslinks were mapped to simulated structural models of (a) apo DGAT1 and (b) lipid-bound DGAT1 based on the reported structure (PDB: 6VYI). Notably, the K391–K224 crosslink was observed only in lipid-bound DGAT1, suggesting a lipid-induced conformational change that brings these residues into closer spatial proximity. K53 is not resolved in the structure; therefore, the corresponding crosslinks cannot be mapped. Molecular docking indicates two potential PA-binding sites, corresponding to a surface cluster and a chamber cluster. The distances between PA in the surface cluster and residues K309 and K312 are 10.9 Å and 5.8 Å, respectively, whereas the corresponding distances in the chamber cluster are 17.8 Å and 14.8 Å.

### Structural Insights from Molecular Docking Analysis of Azi-PA in DGAT1

To visualize lipid-binding modes consistent with the MS-defined crosslinking sites, Azi-PA was docked into DGAT1 using a 30 Å cubic box centered on K309 and K312 within the TM5-TM6 loop (Figure S31). Docking yielded five representative poses that segregated into two recurrent binding regions: a surface cluster and a chamber cluster (Figure 4). Importantly, both clusters are compatible with the experimentally observed dual modification of K309 and K312, supporting the possibility that multiple PA molecules can be accommodated within this region, consistent with LC–MS/MS evidence of two lipid-attached states.

Residue-level interaction analysis further distinguishes these two binding modes. The surface cluster engages ∼30 residues and maps to an exposed luminal pocket centered on the TM5–TM6 loop surrounding K309 and K312 (Table S1). In contrast, the chamber cluster involves ∼37 residues and positions PA deeper within a membrane-proximal cavity adjacent to the catalytic region, spanning helices TM4–TM7 and TM9. Notably, both clusters share a common contact residue, F311, suggesting a structurally related but spatially distinct binding region.

In the surface cluster, PA–K312 distances range from 5.8 to 30.4 Å, whereas distances to K309 are slightly longer (10.9–32.2 Å). The broad distance distributions indicate substantial ligand mobility within the surface binding site. In contrast, the chamber cluster exhibits narrower distance distributions, suggesting a more confined binding mode (Figure S31d). The distances between PA and residues K312 and K309 are 14.8 Å and 17.8 Å, respectively, both of which fall within the expected 11–20 Å range for DSS crosslinks, further supporting the experimental observations (Figure 4b).

Notably, the chamber cluster uniquely engages R322, whereas the surface cluster preferentially interacts with R427. Both residues were included in the M2 mutagenesis set, and mutations at these positions significantly reduced TAG synthesis, providing functional validation of both predicted binding sites.

## Conclusion

We have developed Azi-LPXL-MS as a chemically defined, site-resolved strategy for interrogating lipid–protein interactions. By leveraging catalytic installation of an N–H aziridine handle onto lipid C=C double bonds, this approach converts native lipids into selectively addressable crosslinking partners. This approach enables simultaneous capture of lipid–protein and intraprotein crosslinks in a single workflow, thereby providing localization of lipid-binding sites, spatial constraints between lipids and proteins, structural characterization of bound lipids, and insights into lipid-induced conformational dynamics. Application to BLG establishes accurate residue-level localization of lipid binding and ligand-induced structural changes while additionally providing solution-phase and conformational information not accessible from static structures alone. Extension to DGAT1 demonstrates the applicability of this strategy to complex and structurally dynamic membrane proteins, enabling identification of site-specific PA interactions and associated conformational changes, including in regions not resolved by conventional structural methods such as crystallography and cryo-EM. Integration with site-directed mutagenesis and molecular docking, two potential PA-binding sites in DGAT1, located in a surface pocket and an internal chamber, were identified and functionally validated, further supporting the experimental findings. Although the present study demonstrates successful characterization of membrane-associated lipid interactions in DGAT1, the applicability of Azi-LPXL-MS to deeply buried lipid-binding sites within transmembrane domains remains to be established. While the membrane permeability of DSS suggests that crosslinker accessibility may not be the primary limitation in membrane environments, successful crosslink formation is likely influenced by both the conformational behavior of the aziridinated lipid and the availability of suitably positioned lysine residues. Within this defined scope, the current results demonstrate the utility of Azi-LPXL-MS for interrogating membrane-associated lipid–protein interactions. These results establish Azi-LPXL-MS as a powerful method for resolving lipid–protein interfaces at site resolution and linking lipid binding to protein structural and functional outcomes in complex biological systems.

## Supplementary Material





## Data Availability

The raw mass spectrometry data associated with the crosslinking experiments have been deposited in Zenodo and are publicly available at 10.5281/zenodo.20839213.

## Notes

Information on crosslinks identified in BLG and DGAT1 (XLSX)
Experimental methods; aziridinated lipid characterization data; BLG crosslinking optimization studies; denatured BLG crosslinking results; validation of crosslinks by Cα–Cα distance analysis; POPA-treated DGAT1 XL-MS control experiments; and additional MS spectra and molecular dynamics simulation data­(PDF)
