# Supplementary material for: Aziridination-Based Crosslinking Enables Site-Resolved Elucidation of Lipid Binding and Protein Conformational Changes
Source: J Am Chem Soc. 2026 Oct 7;148(41):44070–8. doi: 10.1021/jacs.6c08027 (PMC13693381; doi:10.1021/jacs.6c08027)
Supplement: Supplementary file 1 [file ja6c08027_si_002.pdf]

Supporting Information

Aziridination-based crosslinking enables site-resolved elucidation of lipid binding and protein conformational changes

Chia-Lung Tsai[a], Atiya Tahira Tasnim[b], Zhenyu Xi[a], Syuan-Ting Kuo[a], Gopal Reddy Ramidi[a], Jiaxin Feng[a], Michael Ryan Eastabrook[a], David H. Russell[a], Xuewu Sui[b], Xin Yan[a, *]

[a] Department of Chemistry, Texas A&M University, 580 Ross St., College Station, TX 77843, USA

[b] Department of Biochemistry and Biophysics, Texas A&M University, 300 Olsen Boulevard, College Station, TX 77843, USA

Table of Contents

S1. Experimental methods 3

S2. Mass spectra of Azi-OA.. 8

S3. Native mass spectra of BLG and lipid interactions in apo BLG, BLG treated with DSS and treated with Azi-OA and DSS. 9

S4. BLG crosslinking reaction optimization 10

S5. Overlap of the crosslinked residue pairs identified in biological triplicate................ 12

S6. Mass spectra of peptide–peptide crosslinked products in BLG 13

S7. Structural dependence of lipid–protein crosslinking revealed by denatured BLG ... 16

S8. Validation of Crosslinks by Cα–Cα Distance Analysis 18

S10. Mass spectra of peptide–peptide crosslinked products in DGAT1........................ 20

S11. DGAT1-POPA (PA 16:0_18:1) crosslinking result 24

S12. Additional MS/MS Spectral Analysis of Crosslinks.. 27

S13. Simulation supplementary data 33

References 35

S1. Experimental methods

Materials and Reagents

All reagents and solvents were purchased from commercial sources and without further purification. β-Lactoglobulin B from bovine milk, trypsin protease, iodoacetamide (IAA), and dithiothreitol (DTT), $Rh_2(esp)_2$, hydroxylamine O-sulfonic acid were purchased from Sigma-Aldrich (St Louis, MO) and Thermo Fisher Scientific (Waltham, MA). Acetonitrile (ACN), water (H_2O), dichloromethane (CH_2Cl_2), methanol (MeOH), ethyl acetate (EtOAc), and 1,1,1,3,3,3-hexafluoroisopropanol (HFIP) were purchased from Thermo Fisher Scientific (Waltham, MA) and Sigma-Aldrich (St Louis, MO). Oleic acid (OA) and 1-palmitoyl-2-oleoyl-*sn*-glycero-3-phosphate (POPA) were purchased from Avanti Polar Lipids and Nu-Chek-Prep, Inc. (Alabaster, AL).

Synthesis of aziridinated oleic acid (Azi-OA)

To a solution of oleic acid in HFIP were added pyridine (3 eq), hydroxylamine O-sulfonic acid (1.5 eq) and $Rh_2(esp)_2$ (5 mol%). The reaction was stirred at room temperature for 4h and the solvent was removed under reduced pressure. The crude product was purified by flash column chromatography on silica gel (methanol/dichloromethane = 1:9) to afford the desired Azi-OA as a light-yellow oil in 75% yield.

Synthesis of aziridinated POPA (Azi-PA)

To a solution of 1-palmitoyl-2-oleoyl-*sn*-glycero-3-phosphate (POPA) in HFIP were added pyridine (3 eq), hydroxylamine O-sulfonic acid (1.5 eq), and $Rh_2(esp)_2$ (5 mol%). The reaction was stirred at room temperature for 4 h and solvent was removed under reduced pressure. The crude product was purified by flash column chromatography on silica gel (methanol/dichloromethane = 1:9) to afford the desired Azi-PA as a light-yellow oil in 68% yield.

Expression and Purification of Human DGAT1

The expression of human DGAT1 fused with an N-terminal maltose-binding protein (MBP), and a TEV cleavage site in HEK293 GnTi- (ATCC, CRL-3022) cells was conducted according to published protocols using the BacMam system.[1, 2] The only difference was that, in this purification 50mM HEPES, pH 7.5 was used instead of Tris 50mM, pH 7.5. Then MBP-DGAT1 was eluted with 0.05% w/v digitonin (Sigma-Aldrich) added elution buffer and further the protein was purified after TEV protease digestion by gel filtration using Superose 6 column (GE Healthcare) using HEPES buffer (50mM HEPES, pH 7.5, 400mM NaCl, 10mM $MgCl_2$) supplemented with 0.02% w/v digitonin. The DGAT1 dimer peak fraction was then concentrated up to 25 μM for further XLMS study.

Expression and Isolation of Human DGAT1 Microsome in Yeast

To express and isolate human DGAT1 overexpressing microsome in yeast cells, codon optimized human DGAT1 gene was cloned into pDDGFP_LEU2 vector (Addgene plasmid no. 102334) tagged with a modified EGFP at N-terminus of DGAT1. To generate all the codon optimized DGAT1 mutants, the QhickChange mutagenesis kit (Agilent) was used according to the manufacturer's protocol. After cloning, all the constructs were confirmed by DNA sequencing and further transformation and microsome isolation steps were conducted, maintaining the previous protocol.[3] Finally, pelleted membranes were homogenized in ice-cold HSMG buffer and snap frozen in liquid nitrogen for further activity studies. Membrane solution was also analyzed by SDS-PAGE and Western blot with DGAT1 primary antibody for quantification purposes.

Human DGAT1 Activity in Yeast Microsome

The activity assay of wild-type DGAT1 or mutants was performed following the previous protocols with slight variation.[3] In brief, to assess the activity of DGAT1 wild-type and its mutant in the presence of PA, 100μg isolated DGAT1 microsomes were incubated with 100 μM POPA at 37 °C, 500 rpm for 30 minutes. Then the mixture was supplemented with 100 μM DAG and 50 μM oleoyl CoA containing 0.02 μCi [14C]-oleoyl-CoA as a radioactive tracer to start the enzymatic reaction.

Azirindinated lipid–protein crosslinking reaction

Lipid–Protein Crosslinking of BLG

Lyophilized BLG was reconstituted in PBS buffer (pH 7.4) at a concentration of 0.5–1.0 mg/mL. BLG was incubated with aziridinated oleic acid (Azi-OA) at a molar ratio of 1:5 (BLG:Azi-OA) for 30 min at room temperature to allow lipid–protein interactions. DSS was subsequently added at a molar ratio of 1:5 relative to BLG (BLG:DSS = 1:5, mol/mol), and the mixture was incubated for an additional 1 h at room temperature. The reaction was quenched with 100 mM ammonium bicarbonate or 200mM ammonium acetate prior to MS analysis.

Lipid–Protein Crosslinking of DGAT1

Purified DGAT1 in HEPES buffer containing 0.02% glyco-diosgenin (50 mM HEPES, pH 7.5, 200 mM NaCl, and 10 mM $MgCl2$) was incubated with aziridinated phosphatidic acid (Azi-PA) at a molar ratio of 1:20 (DGAT1:Azi-PA) for 30 min at room temperature to allow lipid–protein interactions. DSS was then added at a molar ratio of 1:5 relative to DGAT1 (DGAT1:DSS = 1:5, mol/mol), followed by incubation for an additional 1 h at room temperature. The reaction was quenched with 100 mM ammonium bicarbonate or 200mM ammonium acetate prior to MS analysis.

Protein digestion

The crosslinked proteins were buffer-exchanged into 8 M urea in 100 mM ammonium bicarbonate containing 5 mM DTT and incubated at 50 °C for 45 minutes to denature the proteins. Subsequently, iodoacetamide was added for cysteine alkylation and the mixture was incubated at room temperature for 30 minutes in a dark environment. The reaction was then quenched by the addition of DTT and incubated for 15 minutes. The sample was further buffer-exchanged into 100 mM ammonium bicarbonate containing 1 mM $CaCl_2$ and 5 mM DTT. Trypsin (trpsin/protein=1/25) was added, and the mixture was incubated at 37 °C for 16 hours for digestion. The digestion was quenched by adding 50% formic acid to a final concentration of 5%. The resulting peptides were analyzed by HPLC-MS/MS.

Native mass spectrometry

The total concentration (determined by A280) of crosslinked protein was 10 μM. Crosslinked protein was buffer exchanged to 200 mM ammonium acetate with a 7k cut-off spin column (Zeba™ Spin Desalting Columns) prior to mass spectrometry analysis. Native mass spectrometry was carried out on a Q-Exactive UHMR (Thermo Fisher Scientific) with Nanospray Flex ion source (Thermo Fisher Scientific). Spray voltage was set to 1.0 – 1.5 kV and the temperature of the ion transfer tube was set to 50°C. To remove the non-specific adducts, activation energies were set to in-source dissociation (CID): 10 V, higher energy in collision cell (HCD): 30V, and trapping gas pressure: 3 (arbitrary unit). The resolution was set to 6250.

HPLC-MS/MS

The digested peptides were injected into a Vanquish HPLC system (Thermo Fisher Scientific) equipped with a C18 column (TARGA C18, 3 μm, 50 × 1.0 mm I.D.) at a flow rate of 0.1 mL/min. Peptides were eluted using a linear gradient: 0% to 50% solvent B over 30 minutes, followed by an increase to 95% B over 20 minutes. Solvent A consisted of water with 0.1% formic acid (FA), and solvent B consisted of acetonitrile (ACN) with 0.1% FA. Separated peptides were directly introduced into an Orbitrap Fusion mass spectrometer (Thermo Fisher Scientific). MS1 and MS2 spectra were acquired in the Orbitrap at mass resolutions of 120,000 and 15,000, respectively. The MS1 scan range was set from *m/z* 300 to 2,000, with standard automated gain control (AGC) target, standard maximum injection time, and a dynamic exclusion of 30 s. Only precursor ions with charge states from +2 to +7 and intensities above 1.0×10^4 were selected for MS2. For MS2 acquisition, the AGC target was set to 150, with a maximum injection time of 500 ms and an isolation window of 2.0 m/z. Peptide fragmentation was performed using HCD with a normalized collision energy of 30%. Data were acquired in a top-20 data-dependent acquisition mode.

Native MS data analysis

Native MS data were deconvoluted using UniDec.[4] Deconvolution was performed over a mass range of 5-500 kDa with a charge-state range of 1-50 and a mass sampling interval of 10 Da. Peak detection was carried out using a 50 Da window and an intensity threshold of 0.1. All other parameters were kept at their default values.

HPLC-MS data analysis

Mass spectrometry data were analyzed using the Sequest HT search engine for standard peptide and lipid crosslinking site identification and the XlinkX node for lysine-crosslinked peptide identification within Proteome Discoverer (PD; version 3.2; Thermo Fisher Scientific). The Sequest HT search parameters settings were used: MS1 precursor ion mass tolerance: 5 ppm.; MS2 fragment ion mass tolerance: 0.06 Da; maximum number of missed cleavages: 2; minimum peptide length: 4; maximum peptide length: 150.; static modification: Cys: carbamidomethylation (+57.021 Da); dynamic modification: Met: oxidation (+15.995 Da), Lys: DSS (+138.068 Da), Hydrolyzed DSS (+156.079 Da) and Azi-OA with DSS (+435.335 Da)/Azi-PA with DSS (+827.568 Da; neutral loss:-392.473, -$C_{19}H_{37}O_6P$); Peptide spectrum matches (PSMs) were filtered using a false discovery rate (FDR) of 1%. The XlinkX node parameters settings were used: MS1 precursor ion mass tolerance: 10 ppm.; MS2 fragment ion mass tolerance: 20 ppm; maximum number of missed cleavages: 3; minimum peptide length: 5; static modification: Cys: carbamidomethylation (+57.021 Da); dynamic modification: Met: oxidation (+15.995 Da), Lys: DSS (+138.068 Da) and Hydrolyzed DSS (+156.079 Da); Crosslink spectrum matches (CSMs) were filtered using a false discovery rate (FDR) of 1%. Crosslinked peptides with XlinkX scores ≥40 were considered confidently identified and retained for further analysis. Candidate crosslinks with XlinkX scores <40 were further subjected to manual inspection following the validation guidelines published by Iacobucci and Sinz,[5] including evaluation of precursor mass accuracy, sequence fragment ion coverage, crosslink site localization, and overall spectral quality.

Simulation method

Starting models of human diacylglycerol O-acyltransferase 1 (DGAT1) were generated using AlphaFold 3[6] and aligned to the reference structures 6VYI[1], respectively. Segments with low structural confidence (pLDDT < 70) were rebuilt using MODELLER 10.8[7]. For DGAT1, the disordered N-terminal region (residues 1 to 65) was omitted to reduce computational cost. Ligand parameters were generated with CGenFF[8] through CHARMM-GUI[9].

Molecular docking was performed with AutoDock Vina 1.2[10, 11] using an exhaustiveness of 32 and 50 output poses per pocket. For DGAT1, the docking box was centered on the

COM of the K309/K312 region. From the 50 docked poses generated for each pocket, five structurally diverse poses were selected by greedy maximum-minimum heavy-atom RMSD. Starting from the best-scoring pose, each subsequent pose was chosen to maximize its minimum heavy-atom RMSD to the poses already selected. This procedure was used to retain distinct binding orientations rather than near-duplicate low-energy poses.

All-atom molecular dynamics simulations were performed in GROMACS 2024[12] using the CHARMM36-jul2022 force field[13] and TIP3P water[14]. Each system was placed in a cubic box with 1.5 nm padding, solvated, and neutralized with Na^+ and Cl^- ions. After steepest-descent energy minimization, systems were equilibrated for 500 ps in the NVT ensemble followed by 500 ps in the NPT ensemble, with heavy-atom restraints applied to both protein and ligand. Production simulations were then carried out in the NPT ensemble at 300 K and 1 bar for 20 ns for holo systems and 50 ns for apo systems, using the V-rescale thermostat[15] and Parrinello-Rahman barostat[16]. A 2 fs integration step was used with hydrogen-containing bonds constrained by LINCS[17]. Long-range electrostatics were treated with particle mesh Ewald (PME)[18, 19] using a 1.2 nm real-space cutoff and a 0.16 nm grid spacing. Van der Waals interactions were evaluated with a force-switched cutoff between 1.0 and 1.2 nm. Coordinates were saved every 100 ps.

For each pair of interests, the Euclidean distance between reactive atoms was calculated for every trajectory frame. For protein-protein pairs, the lysine NZ atom was used; for the N-terminal amine, the backbone N atom was used; and for protein-ligand pairs, the diazirine nitrogen was used. Residue-level contact maps were constructed using a 4 Å heavy-atom cutoff over the three frames flanking the snapshot with the minimum pair distance for each pose.

S2. Mass spectra of Azi-OA

(a)

(b)

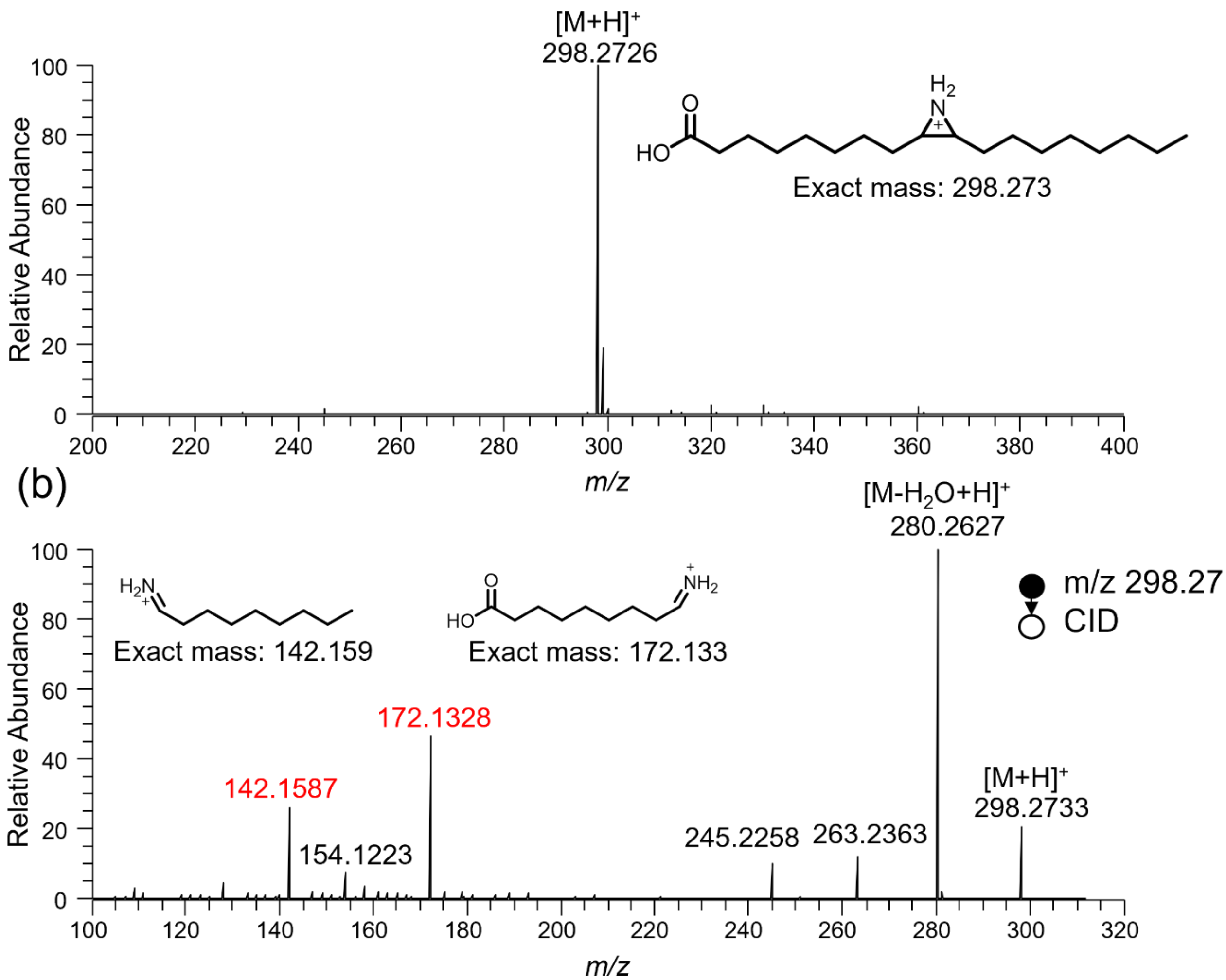

Figure S1. (a) The full mass spectrum and (b) MS2 mass spectrum of Azi-OA, with the diagnostic ion at *m/z* 142.159 and *m/z* 172.133 indicating the double bond position at C_9.

S3. Native mass spectra of BLG and lipid interactions in apo BLG, BLG treated with DSS and treated with Azi-OA and DSS.

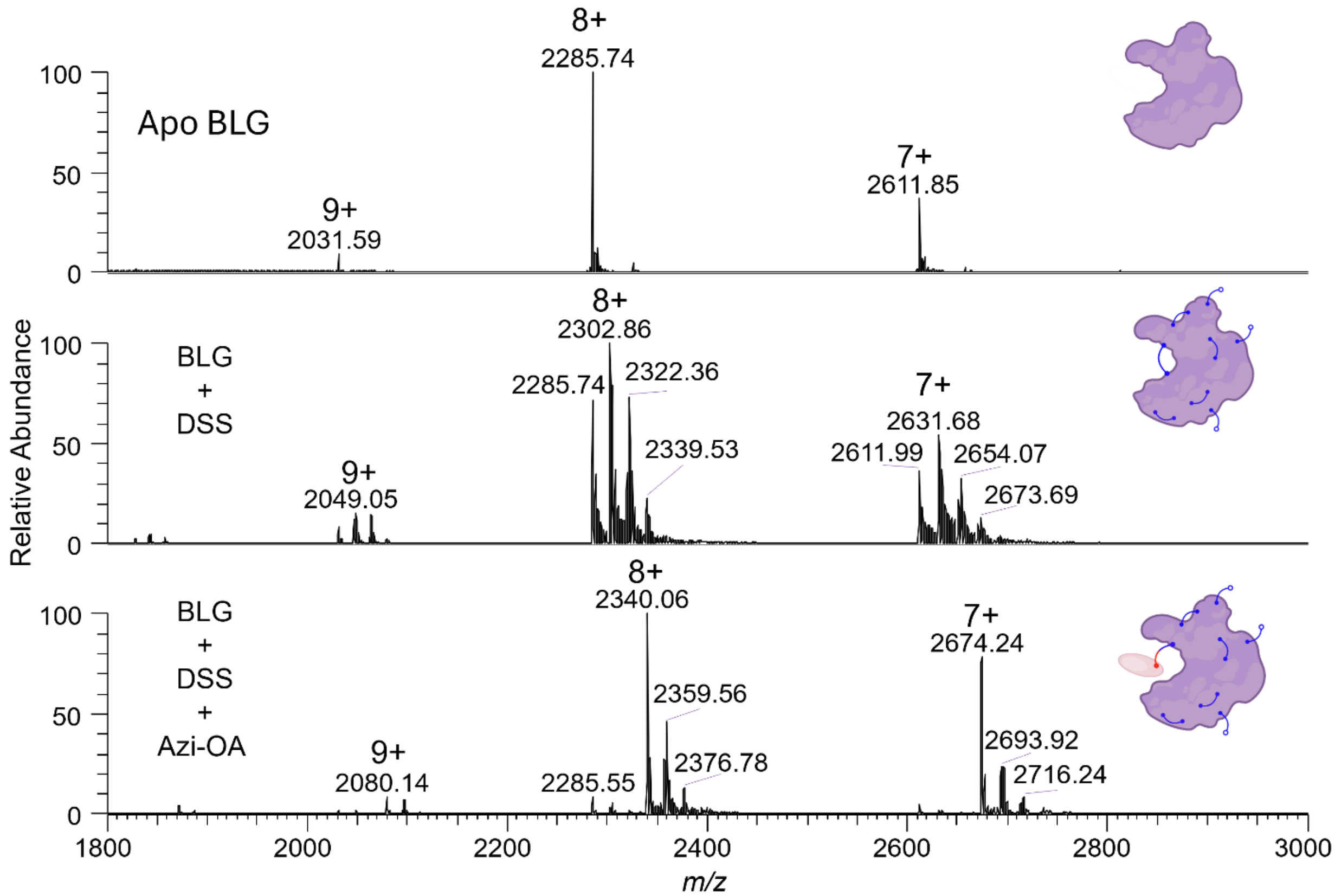

Figure S2. Native mass spectra of BLG and lipid interactions in apo BLG, BLG treated with DSS and treated with Azi-OA and DSS.

Note: Under the optimized crosslinking conditions, native MS predominantly detected species containing a single intraprotein DSS crosslink, although species containing multiple DSS crosslinks were also observed at lower abundance. No significant signals corresponding to BLG dimers were detected. In contrast, bottom-up XL-MS captures the cumulative population of crosslinked proteins present in solution and therefore provides a more sensitive readout for identifying low-abundance crosslinking events.

S4. BLG crosslinking reaction optimization

BLG (20 μM in PBS) was incubated with Azi-OA (dissolved in 1% DMSO in PBS) at room temperature for 30 minutes, allowing BLG to bind with Azi-OA and form a protein–lipid complex. This was followed by the addition of disuccinimidyl suberate (DSS, 100 μM in 10% DMSO PBS buffer), a homobifunctional amine-reactive crosslinker, to perform the crosslinking reaction at room temperature for 1 hour. The reaction was then quenched with 1 mM ammonium acetate, and PBS buffer was replaced by 200 mM ammonium acetate using a 7k cut-off spin column (Zeba™ Spin Desalting Columns) following the standard buffer exchange protocol. To optimize the crosslinking reaction, DSS and Azi-OA equivalents were evaluated separately. BLG first reacted with DSS at varying equivalents (20, 10, 5, 2.5, and 1.25 equiv), along with a no-DSS control, following the standard crosslinking procedure. Native MS analysis showed that increasing DSS equivalents resulted in progressively higher degrees of crosslinking, as evidenced by multiple DSS adducts on the protein (Fig. S3). Prominent crosslinked species corresponding to BLG:DSS ratios of 1:5 to 1:20 were observed. However, higher DSS equivalents (1:10 and 1:20) led to reduced peptide coverage in subsequent bottom-up analysis.[20] Therefore, a BLG:DSS ratio of 1:5 was selected for further crosslinking experiments as a balance between crosslinking efficiency and sequence coverage. Next, the equivalent amount of Azi-OA was optimized at 20, 10, 5, 2.5, and 1.25 equiv, with a fixed DSS ratio of (1:5). Native MS analysis showed that the BLG + DSS + Azi-OA samples exhibited a clear additional mass shift of +435 Da (corresponding to DSS + Azi-OA) at 20, 10, 5, and 2.5 equiv, indicating successful covalent incorporation of Azi-OA into the protein (Figure S4). To ensure sufficient crosslinked product while minimizing nonspecific crosslinking, a BLG:Azi-OA ratio of 1:5 was selected for subsequent analysis. Based on this optimization, a BLG:Azi-OA:DSS ratio of 1:5:5 was selected for subsequent experiments, yielding 58% lipid–protein crosslinking and 21% intraprotein crosslinking. Crosslinking efficiencies were calculated from deconvoluted native mass spectra using UniDec by integrating the signal intensities of each species. Lipid–protein crosslinking efficiency was calculated as the summed intensity of all BLG-Azi-OA adducts (see middle panel in Figure 1b, 18,720 Da, 18,876 Da, and 19,014 Da) divided by the total intensity of all detected species, including apo BLG (18,285 Da) and intraprotein peptide–peptide crosslinked products (18,423 Da and 18,579 Da). Intraprotein crosslinking efficiency was determined analogously from the summed intensities of intraprotein peptide–peptide crosslinked products relative to the same total signal.

$$\text{crosslinking efficiency} = \frac{Sum\ of\ intensities\ of\ BLG\text{-}Azi\text{-}OA\ adducts}{Sum\ of\ total\ intensity\ of\ all\ detected\ species}$$

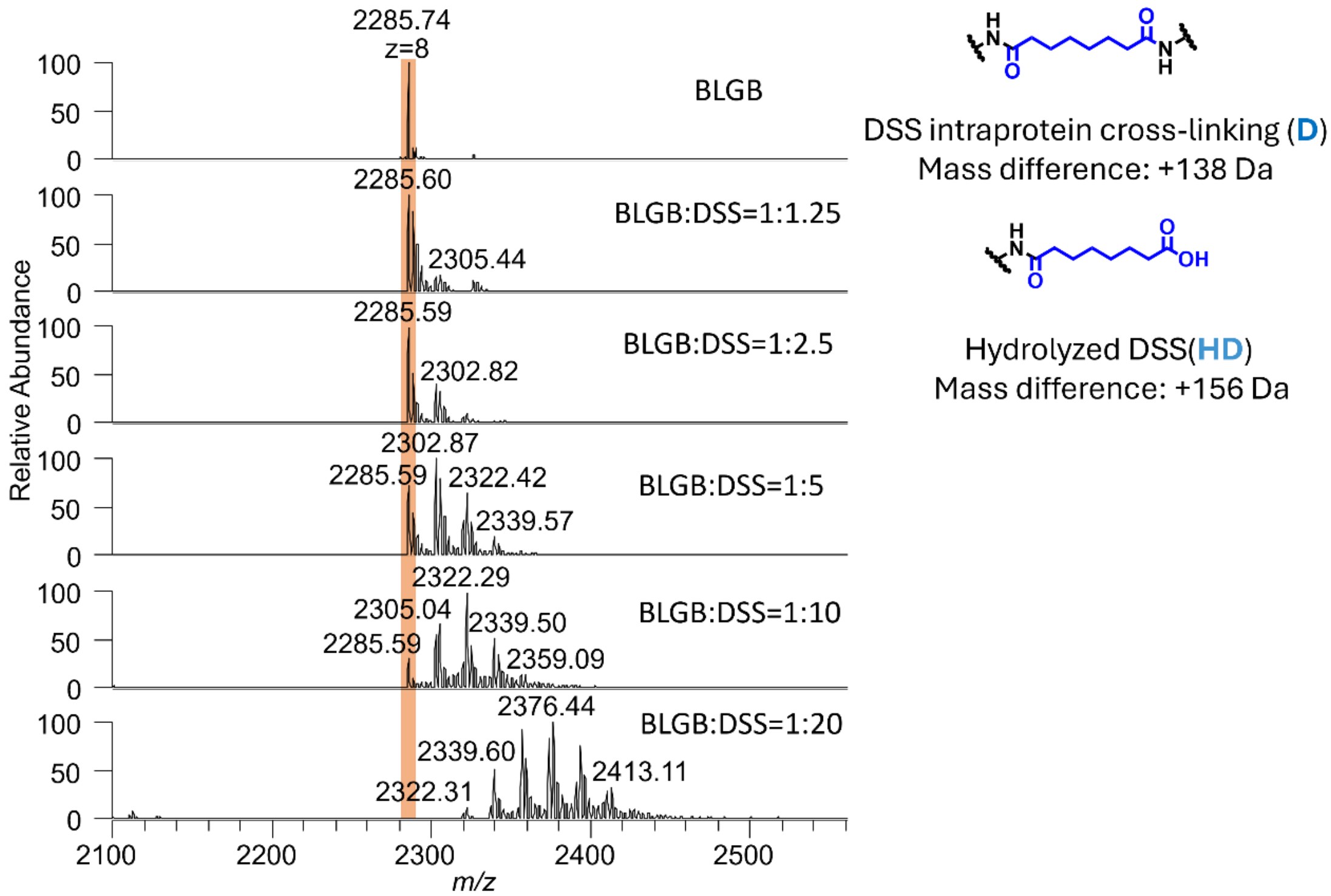

Figure S3. Optimization of DSS equivalents for BLG–Azi-OA crosslinking.

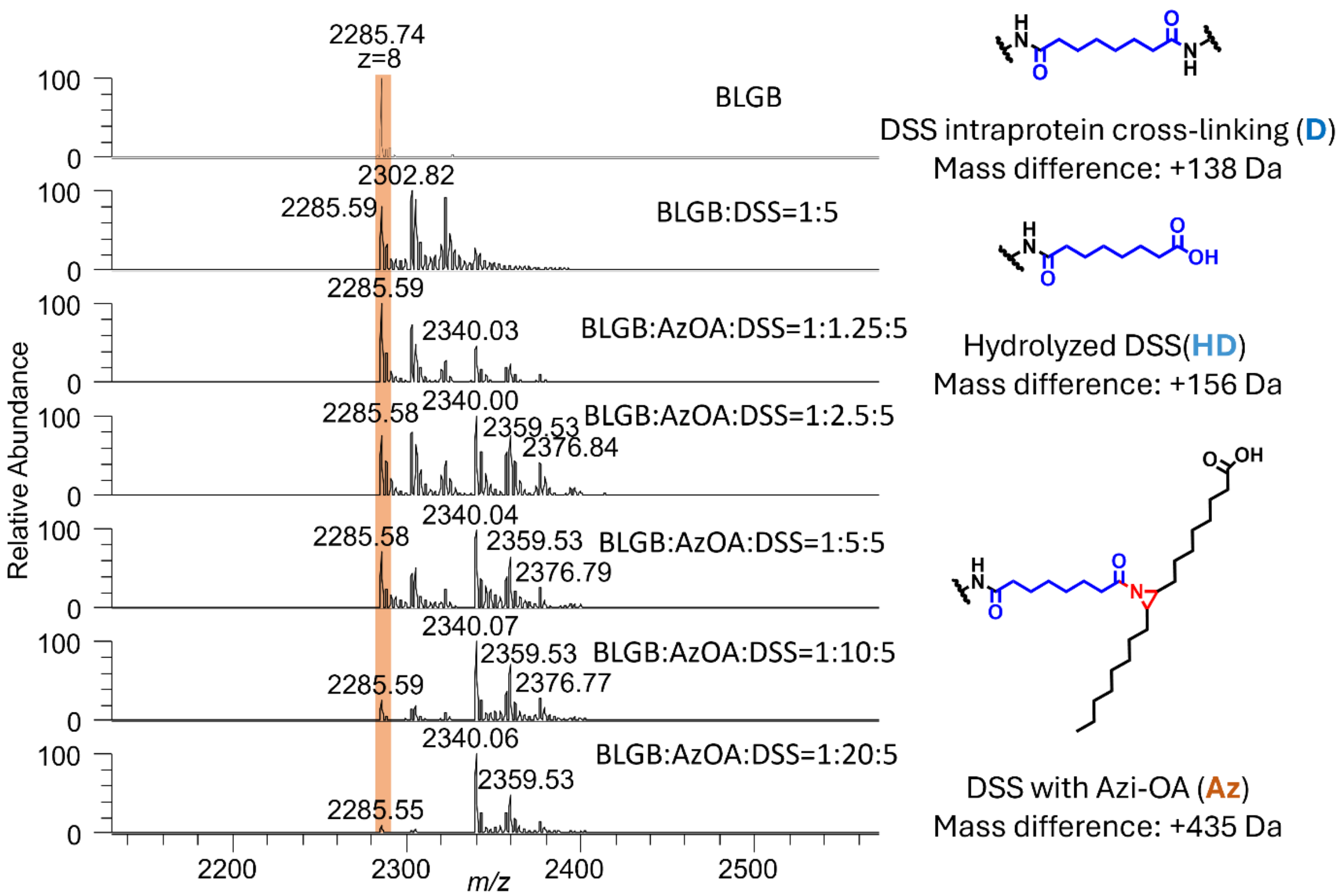

Figure S4. Optimization of Azi-OA equivalents for BLG–Azi-OA crosslinking.

S5. Overlap of the crosslinked residue pairs identified in biological triplicate

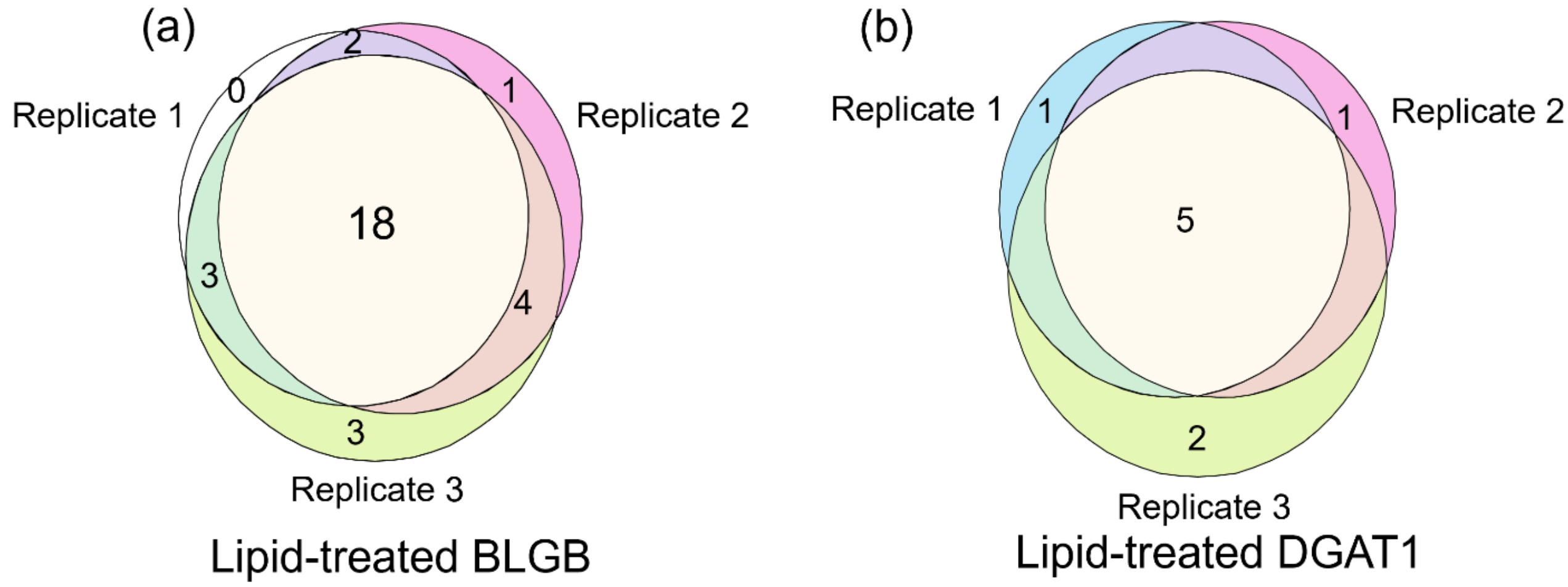

Figure S5. (a) Overlap of crosslinked residue pairs identified across biological triplicates for lipid-treated native BLG. (b) Overlap of crosslinked residue pairs identified across biological triplicates for lipid-treated native DGAT1.

S6. Mass spectra of peptide–peptide crosslinked products in BLG

Apo BLG

Crosslinks are shown only if they passed 1% FDR filtering and were observed in at least 2 of 3 biological replicates. The peptide–peptide crosslinks at K60-K91 and K69-K91 can be found only in apo BLG.

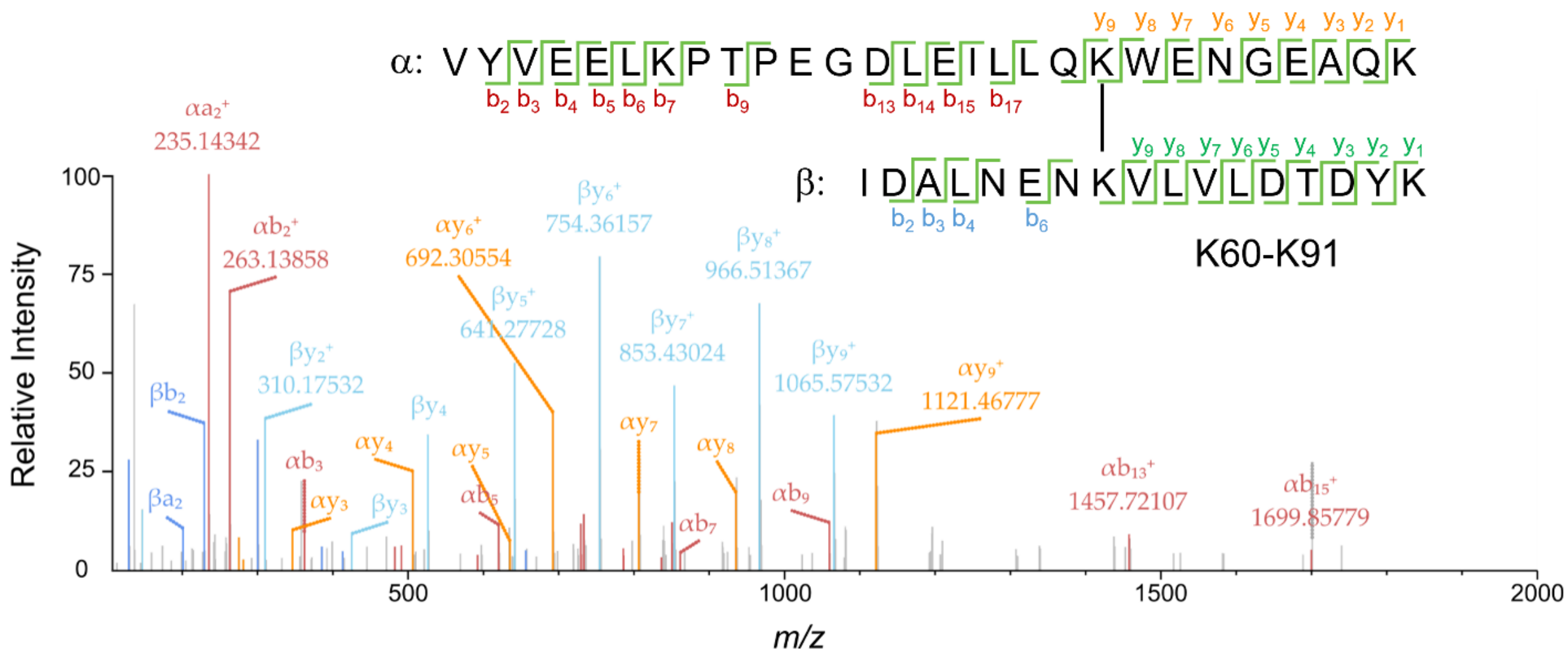

Figure S6. HCD-MS/MS spectrum of peptide–peptide crosslinked products in apo BLG at K60-K91.

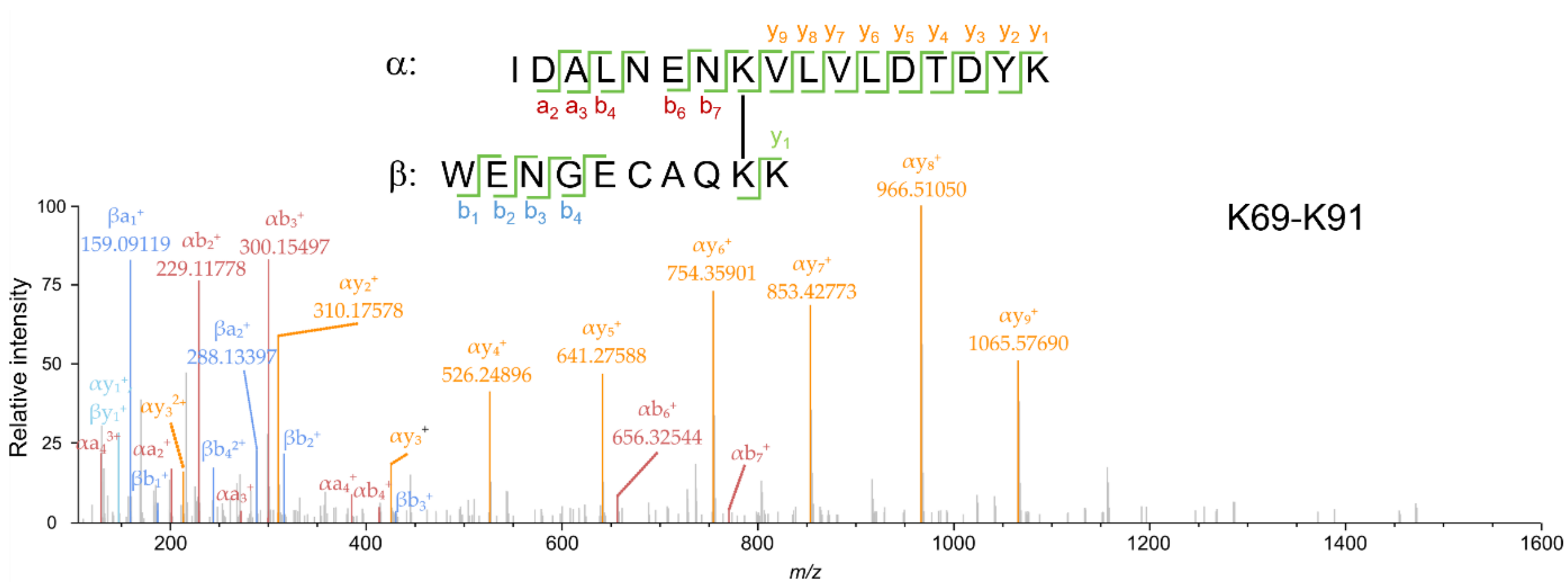

Figure S7. HCD-MS/MS spectrum of peptide–peptide crosslinked products in apo BLG at K69-K91.

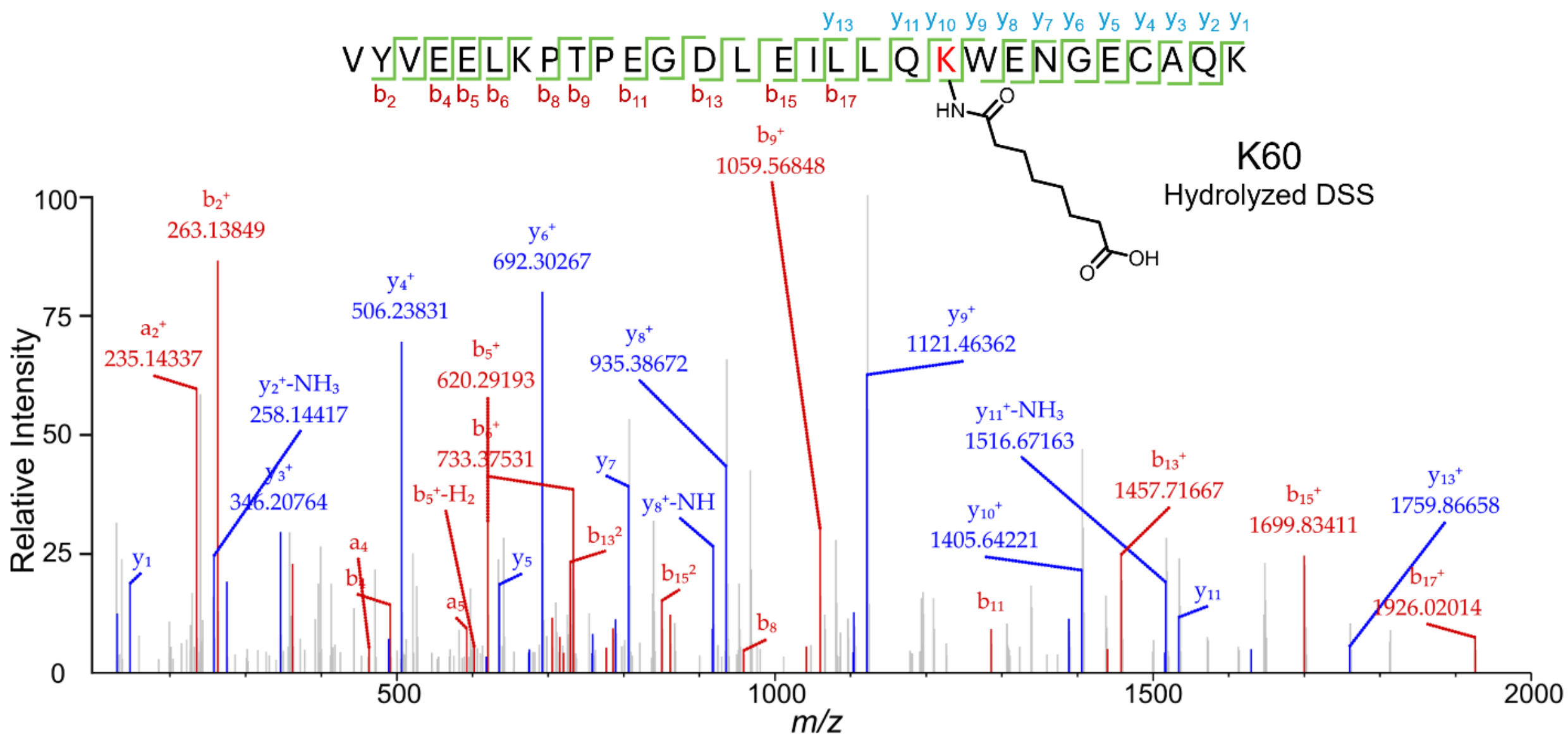

Figure S8. HCD-MS/MS spectrum of peptide–peptide crosslinked products in apo BLG at K60 (Hydrolyzed DSS).

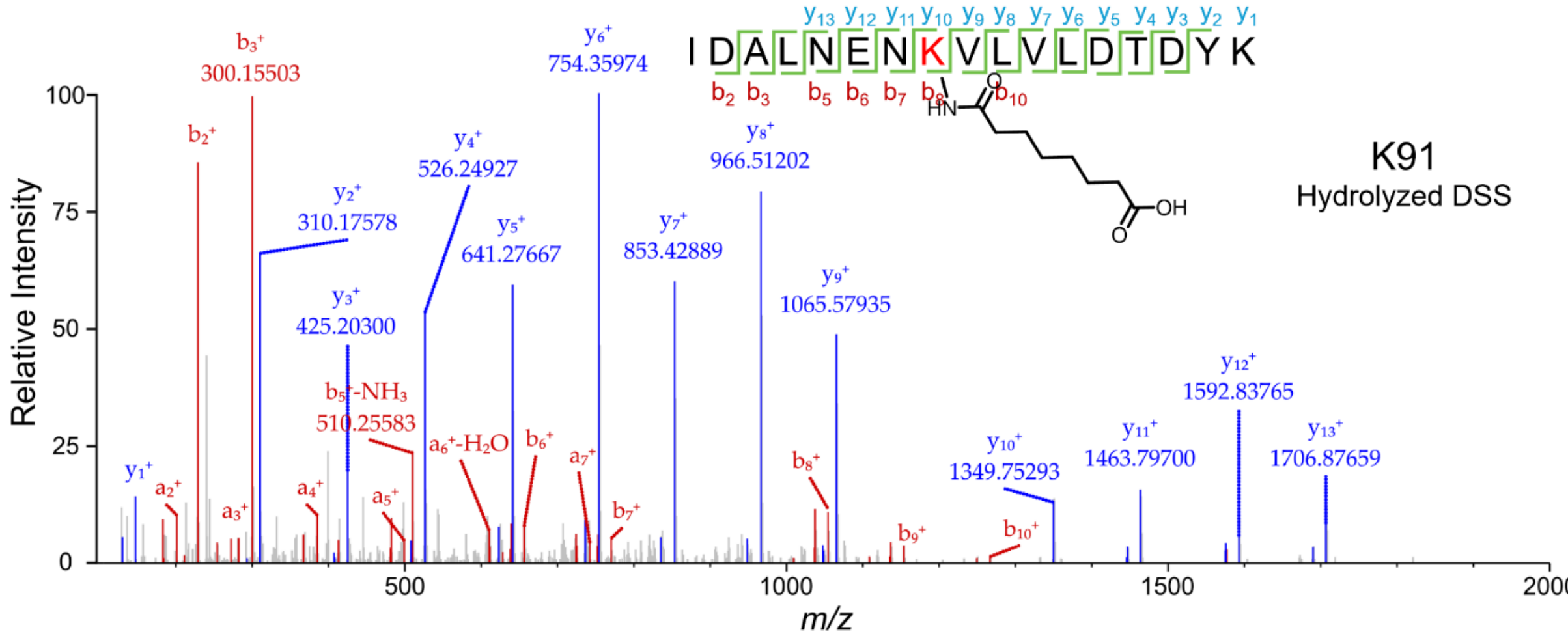

Figure S9. HCD-MS/MS spectrum of peptide–peptide crosslinked products in apo BLG at K91 (Hydrolyzed DSS).

Lipid-treated BLG

Hydrolyzed DSS-modified peptides were detected at K60 and K91 in lipid-treated BLG (K69 is the lipid crosslinking site (Figure 1d)), indicating that DSS can access and react with these residues but is unable to form crosslinks of K60-K91 or K69-K91.

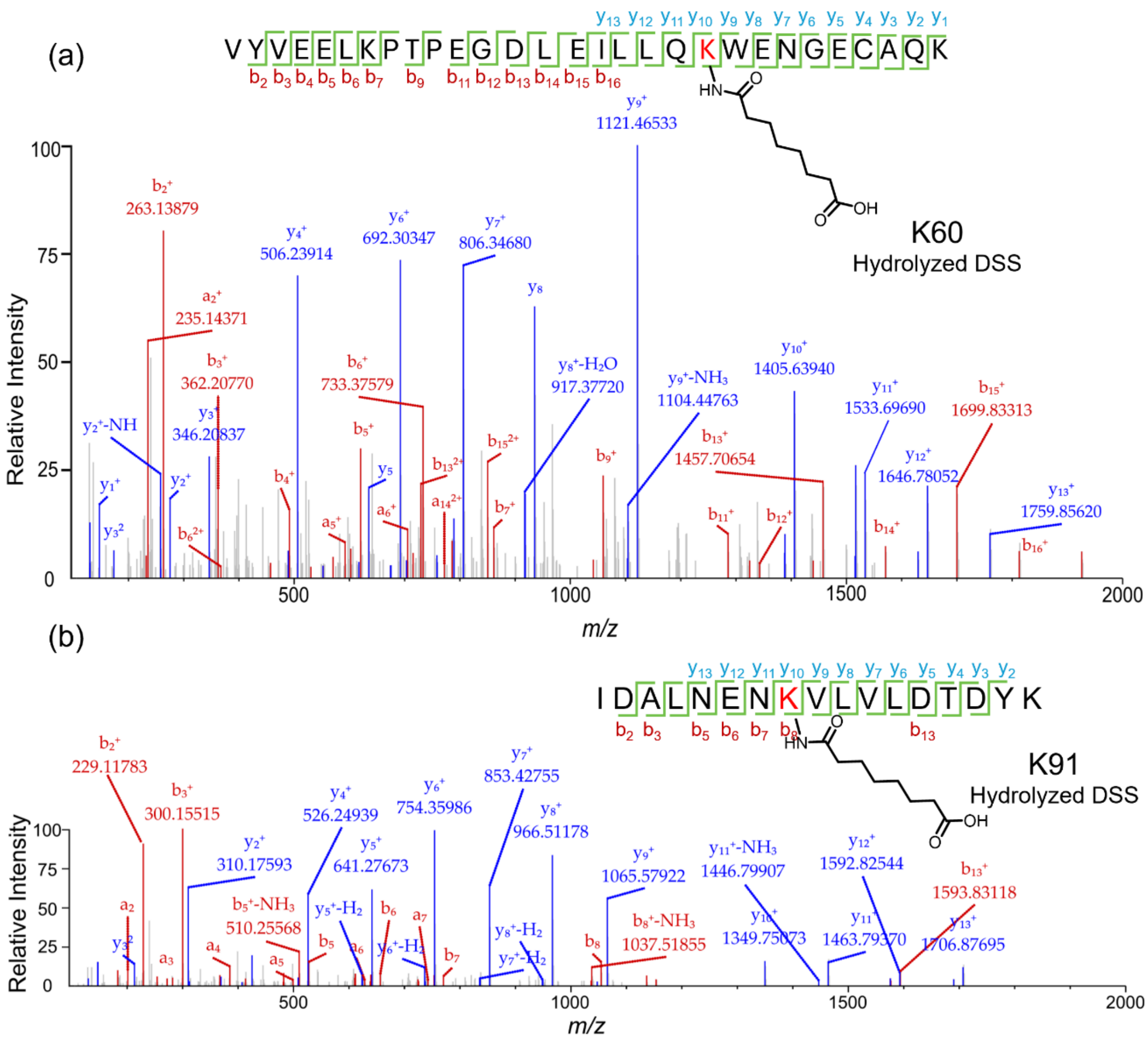

Figure S10. HCD-MS/MS spectra of hydrolyzed DSS at (a) K60 and (b) K91 in lipid-treated BLG. No peptide–peptide crosslinks were detected between K60-K91 or K69-K91.

S7. Structural dependence of lipid–protein crosslinking revealed by denatured BLG

BLG was denatured in 8 M urea prepared in 100 mM ammonium bicarbonate containing 5 mM DTT and incubated at 50 °C for 45 min. Iodoacetamide was then added for cysteine alkylation, and the mixture was incubated at room temperature for 30 min in the dark. The reaction was quenched by the addition of DTT and incubated for a further 15 min. The sample was subsequently buffer exchanged into PBS. The denatured BLG was incubated with Azi-OA for 30 min, followed by DSS addition for 1 h under optimized conditions. The reaction was quenched with 1 mM ammonium acetate and subjected to trypsin digestion. The resulting peptides were analyzed by LC-MS/MS.

Under these conditions, 5 lipid crosslinking sites (K8, K14, K47/K60 and K91) were identified across three biological replicates (Figure S11). Notably, in the absence of a defined tertiary structure, lipid–protein interactions appeared less specific, resulting in more broadly distributed crosslinking sites. Consistent with this observation, 87 and 91 peptide–peptide crosslinks were identified for denatured BLG in the absence and presence of lipid, respectively, exceeding those observed for the native protein. These results suggest that conformational flexibility increases the probability of crosslink formation across multiple lysine residues (Figure S12).

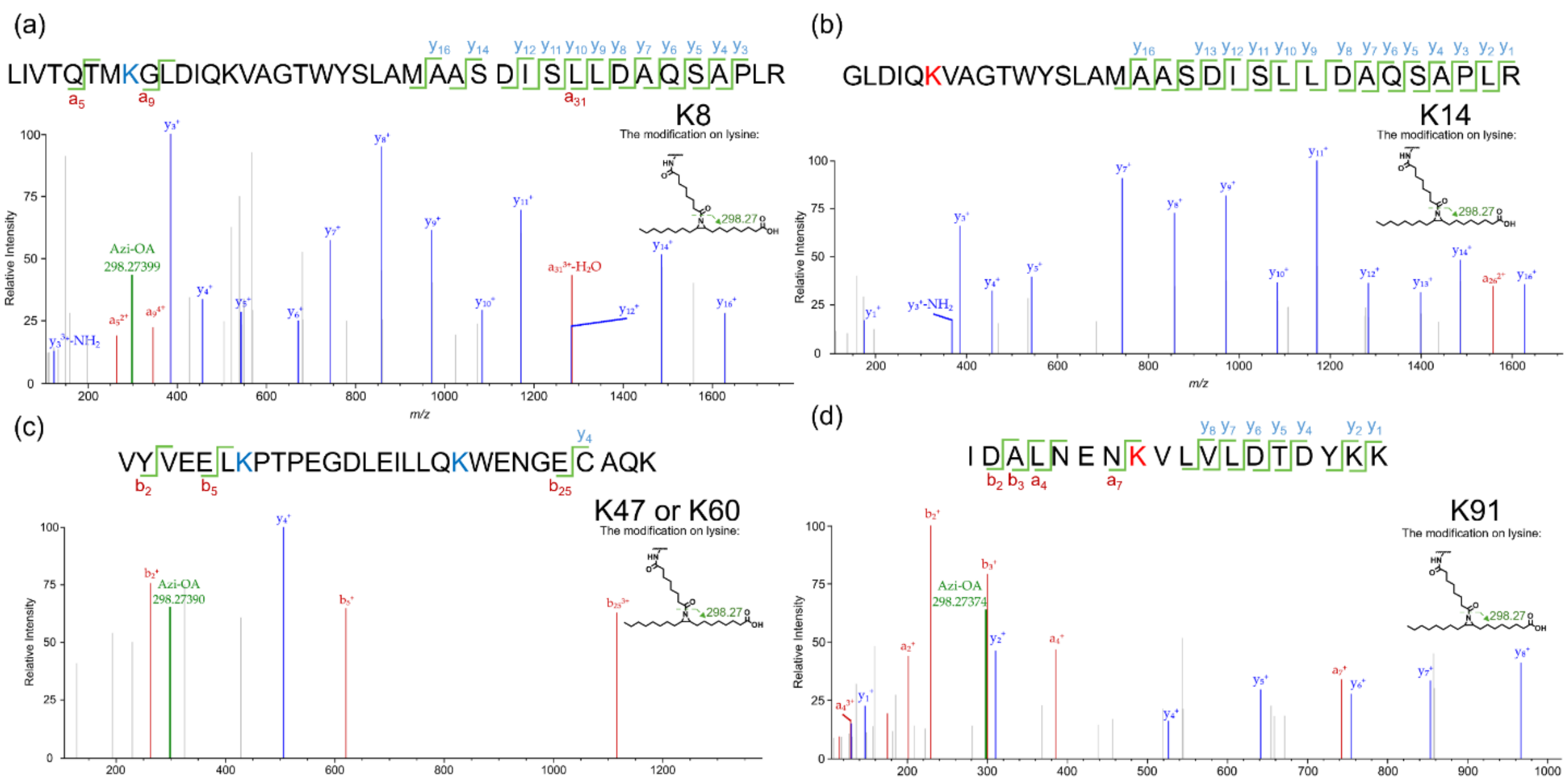

Figure S11. HCD-MS/MS spectra of lipid–protein crosslinked peptides in denatured BLG, identifying crosslinking sites at (a) K8, (b) K14, (c) K47 or K60, and (d) K91.

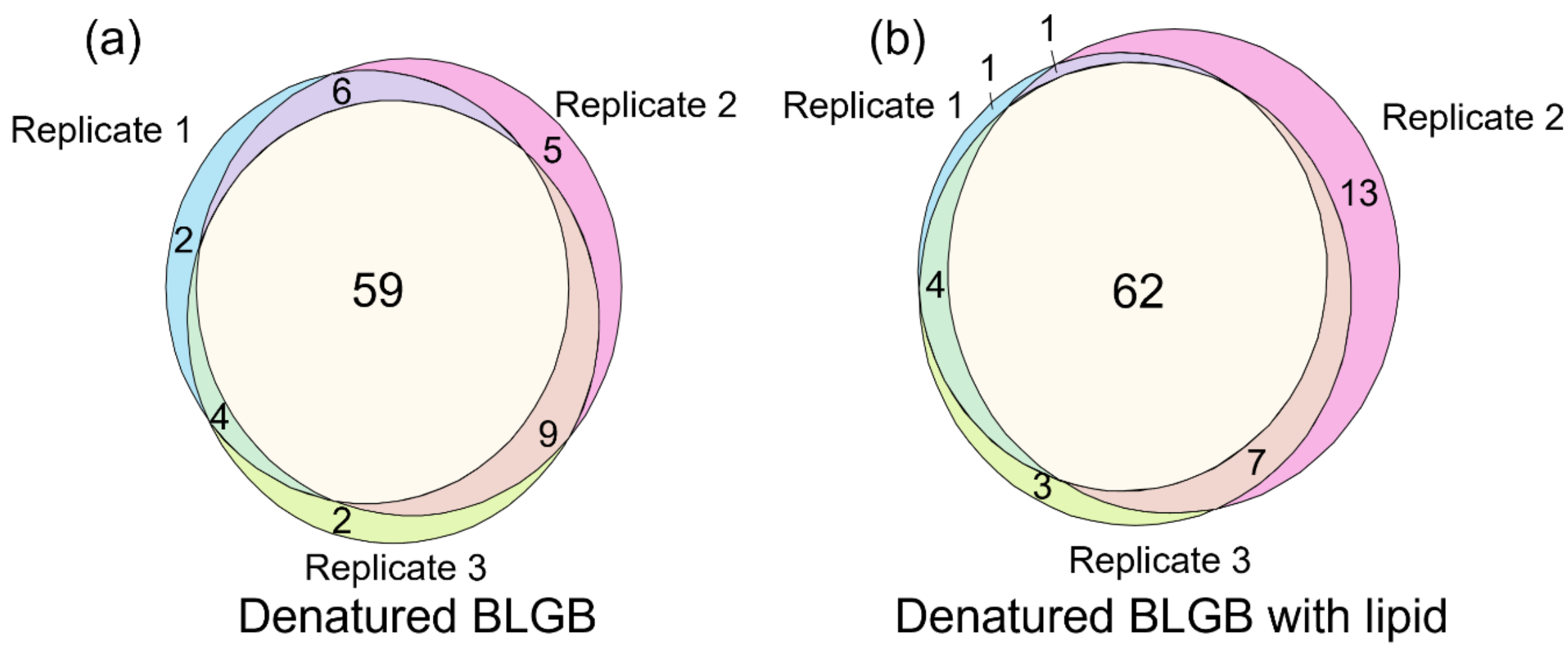

Figure S12. (a) Overlap of crosslinked residue pairs identified across biological triplicates for denatured BLG. (b) Overlap of crosslinked residue pairs identified across biological triplicates for denatured BLG with Azi-OA.

S8. Validation of Crosslinks by Cα–Cα Distance Analysis

To provide an orthogonal structural validation of the identified peptide–peptide crosslinks, the crosslinks were mapped onto the corresponding BLG crystal structures (PDB: 3NPO and 4DQ3), and the Cα–Cα distances between the crosslinked lysine residues were measured. Structural compatibility analysis is widely used in XL-MS because crosslinks are expected to satisfy the geometric constraints imposed by the crosslinker. A conservative Cα–Cα distance cutoff of 26 Å was used for DSS-mediated crosslinking.[21] Using this criterion, 89% of the crosslinks identified in apo BLG and 91% of those identified in lipid-treated BLG satisfied the expected distance constraint. The few crosslinks exceeding the cutoff were only marginally above 26 Å, which is consistent with the conformational flexibility of proteins and the fact that crystal structures represent static conformations. Overall, the structural compatibility analysis provides independent validation of the reported crosslink identifications.

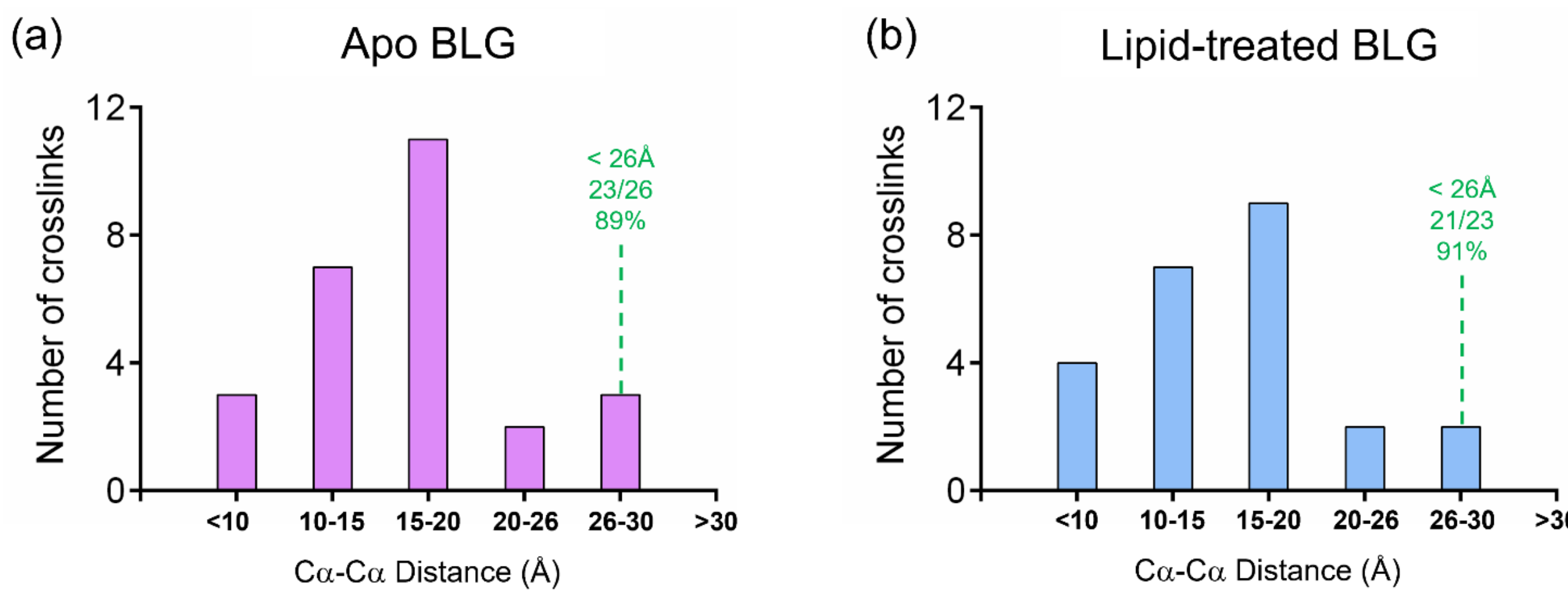

Figure S13. Distribution of Cα–Cα distance of crosslinked residues in (a) Apo BLG and (b) Lipid-treated BLG.

S9. Mass spectra of Azi-PA

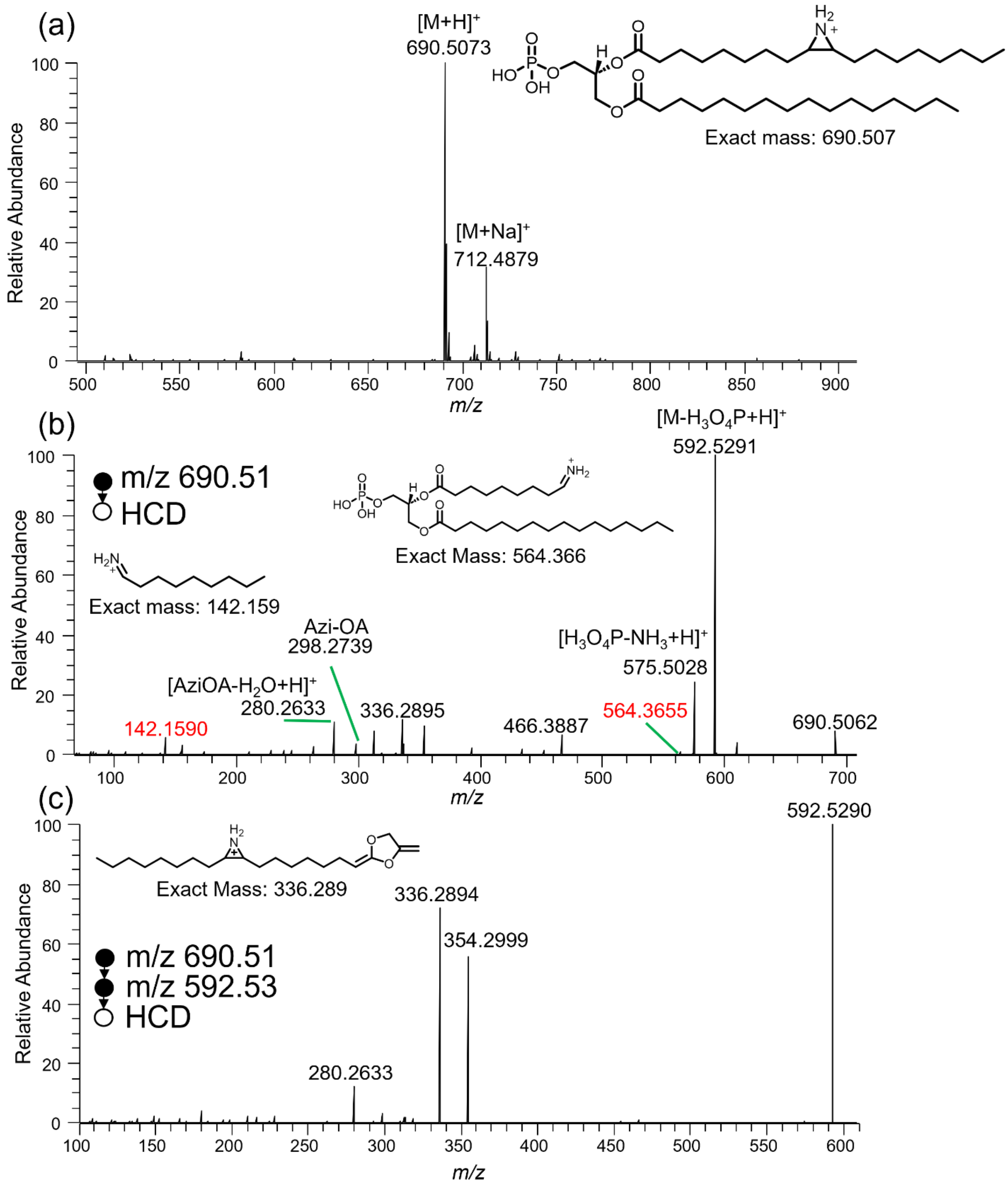

Figure S14. (a) The full mass spectrum, (b) MS2 mass spectrum, and (c) MS3 mass spectrum of Azi-PA, with the diagnostic ions at *m/z* 564.3655 and *m/z* 142.1590 indicating the double bond position at C_9.

S10. Mass spectra of peptide–peptide crosslinked products in DGAT1

Apo DGAT1

Hydrolyzed DSS-modified peptides were detected at K231, K224, and K391 in apo DGAT1, indicating that DSS can access and react with these residues but is unable to form crosslinks of K224-K391 or K54-K231.

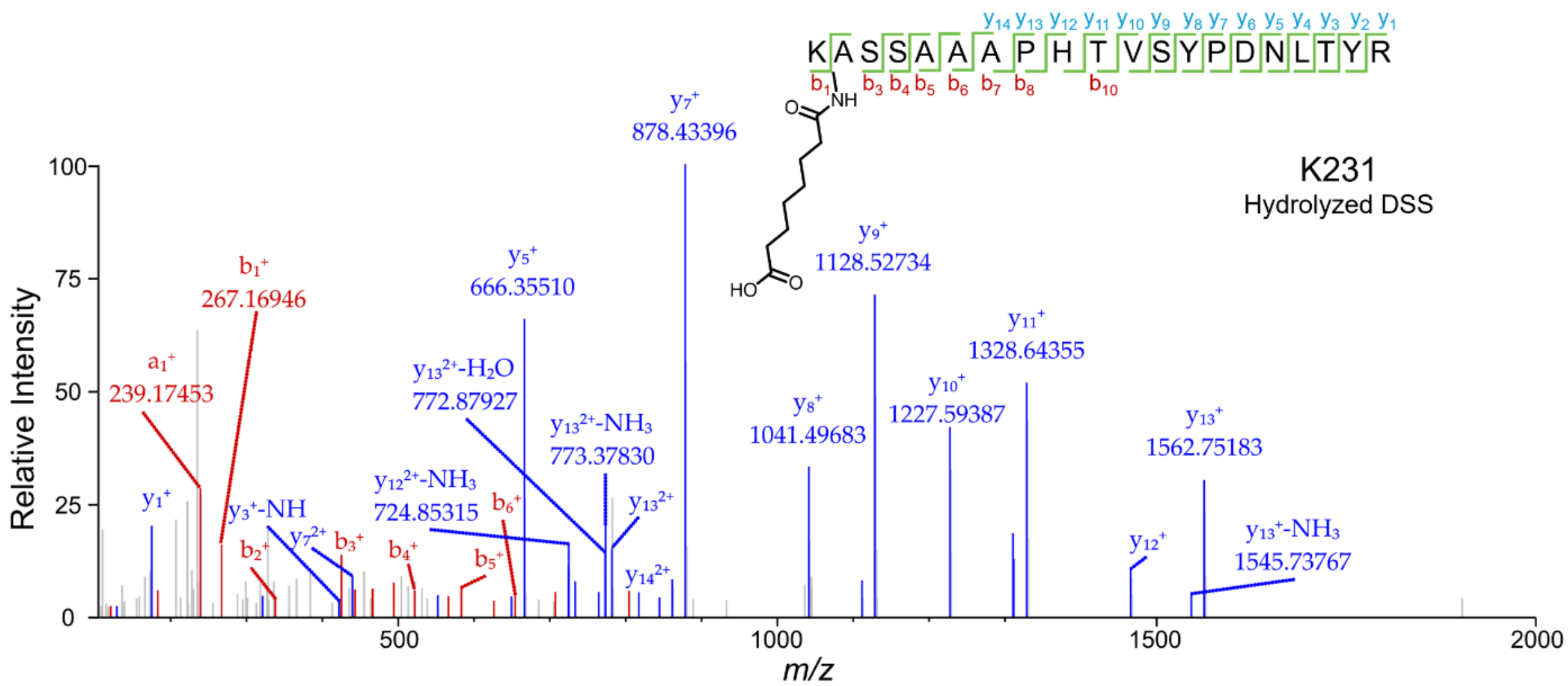

Figure S15. HCD-MS/MS spectrum of hydrolyzed DSS modified peptide at K231 in apo DGAT1.

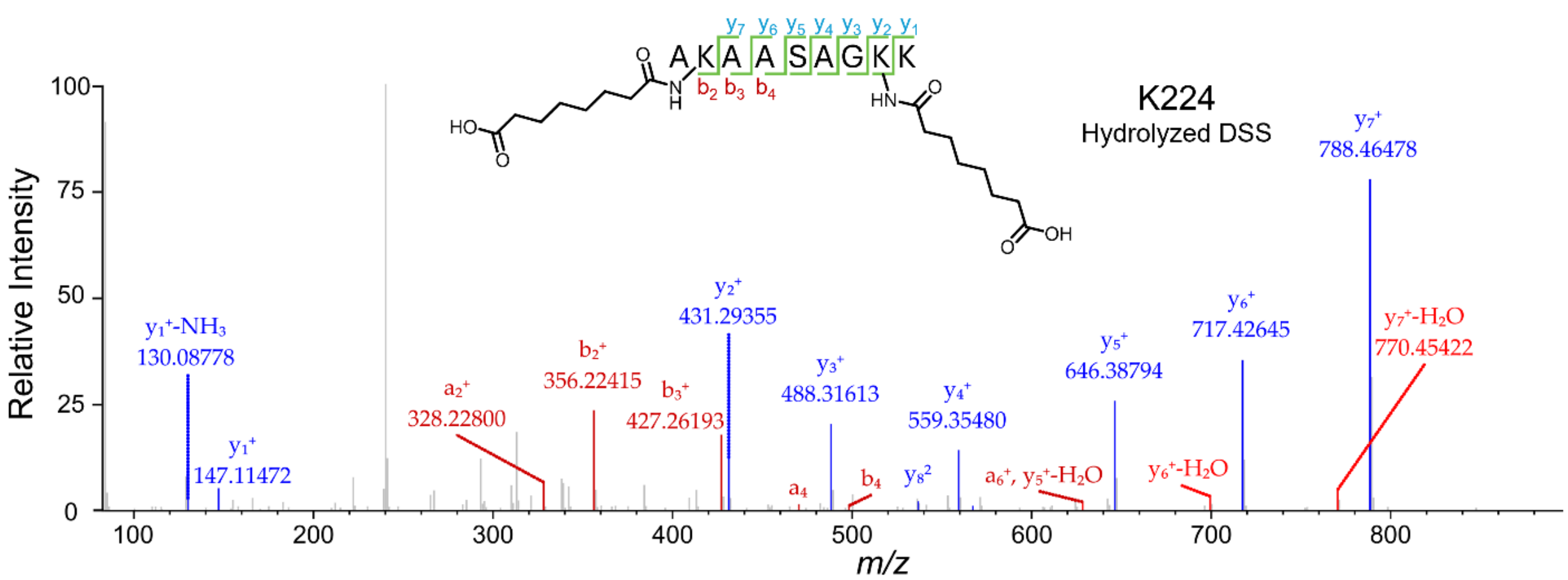

Figure S16. HCD-MS/MS spectrum of hydrolyzed DSS modified peptide at K224 in apo DGAT1.

Lipid-treated DGAT1

Crosslinks are shown only if they passed 1% FDR filtering and were observed in at least 2 of 3 biological replicates. The peptide–peptide crosslinks at K391-K224 and K53-K231 can be found only in lipid-treated DGAT1.

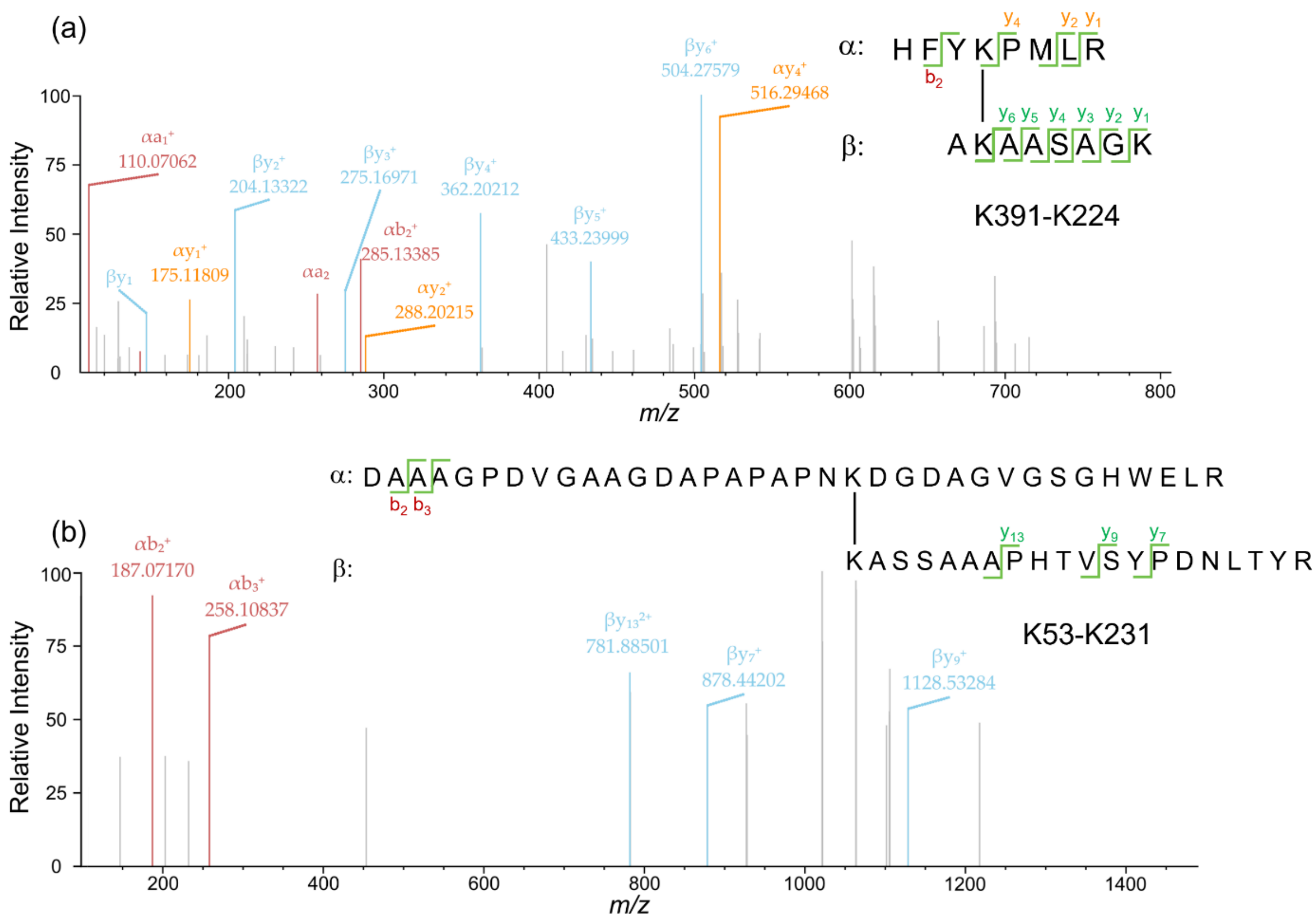

Figure S17. HCD-MS/MS spectra of peptide–peptide crosslinked products in lipid-treated DGAT1 at (a) K391-K224 and (b) K53-K231.

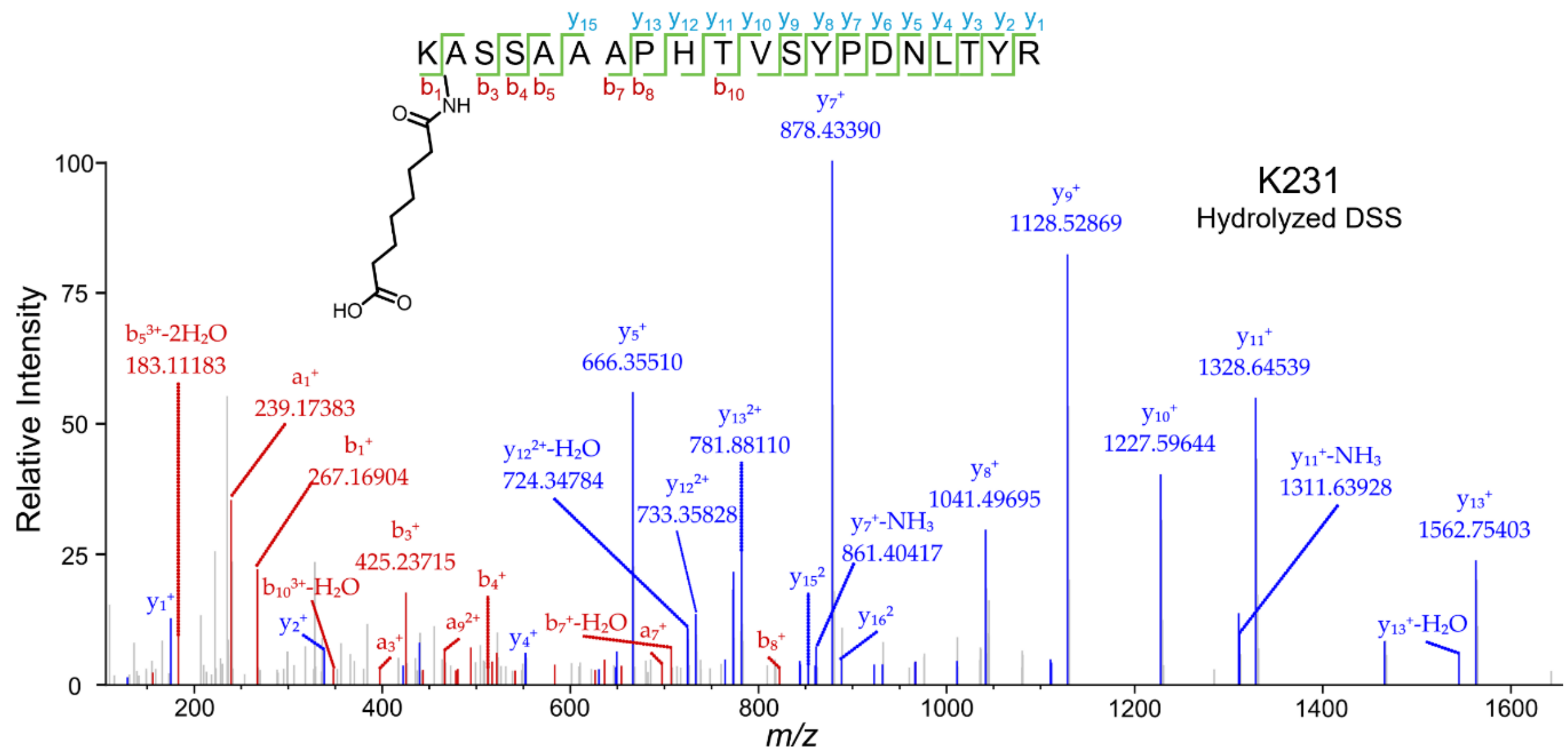

Figure S18. HCD-MS/MS spectrum of hydrolyzed DSS modified peptide at K231 in lipid treated DGAT1.

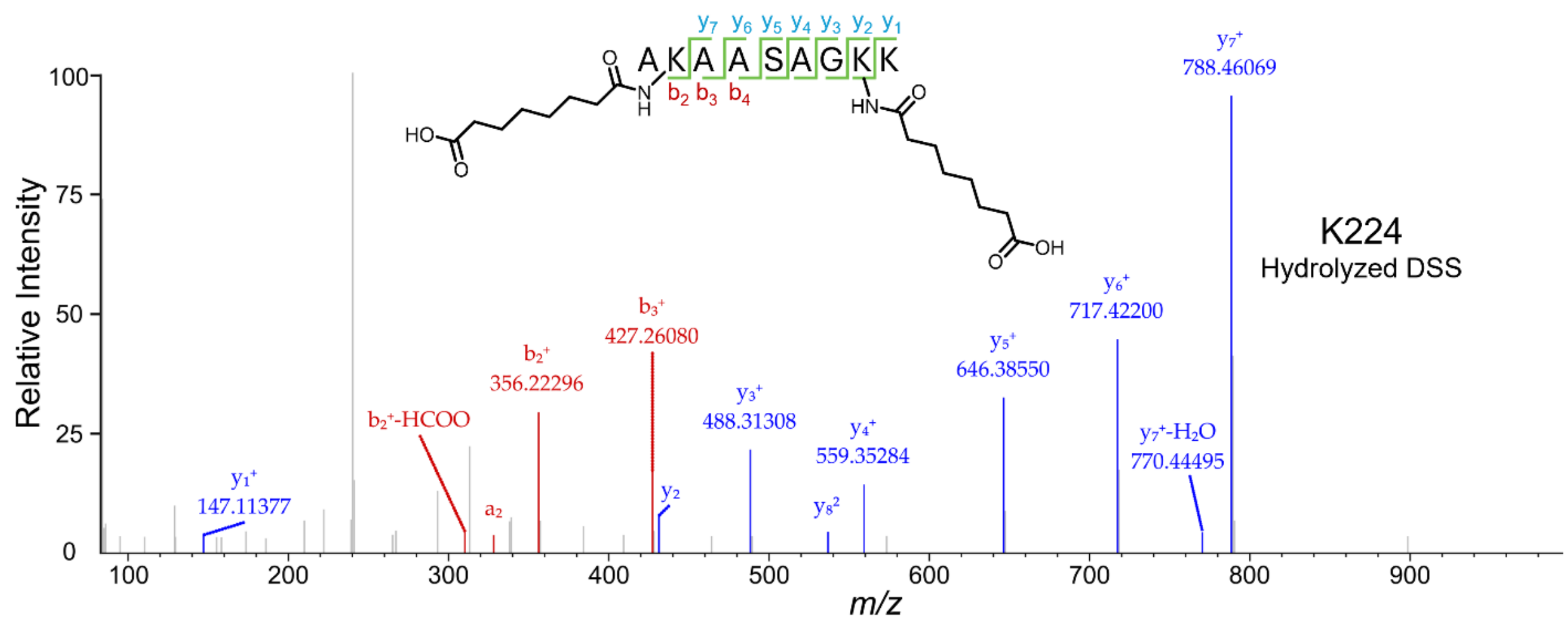

Figure S19. HCD-MS/MS spectrum of hydrolyzed DSS modified peptide at K224 in lipid treated DGAT1.

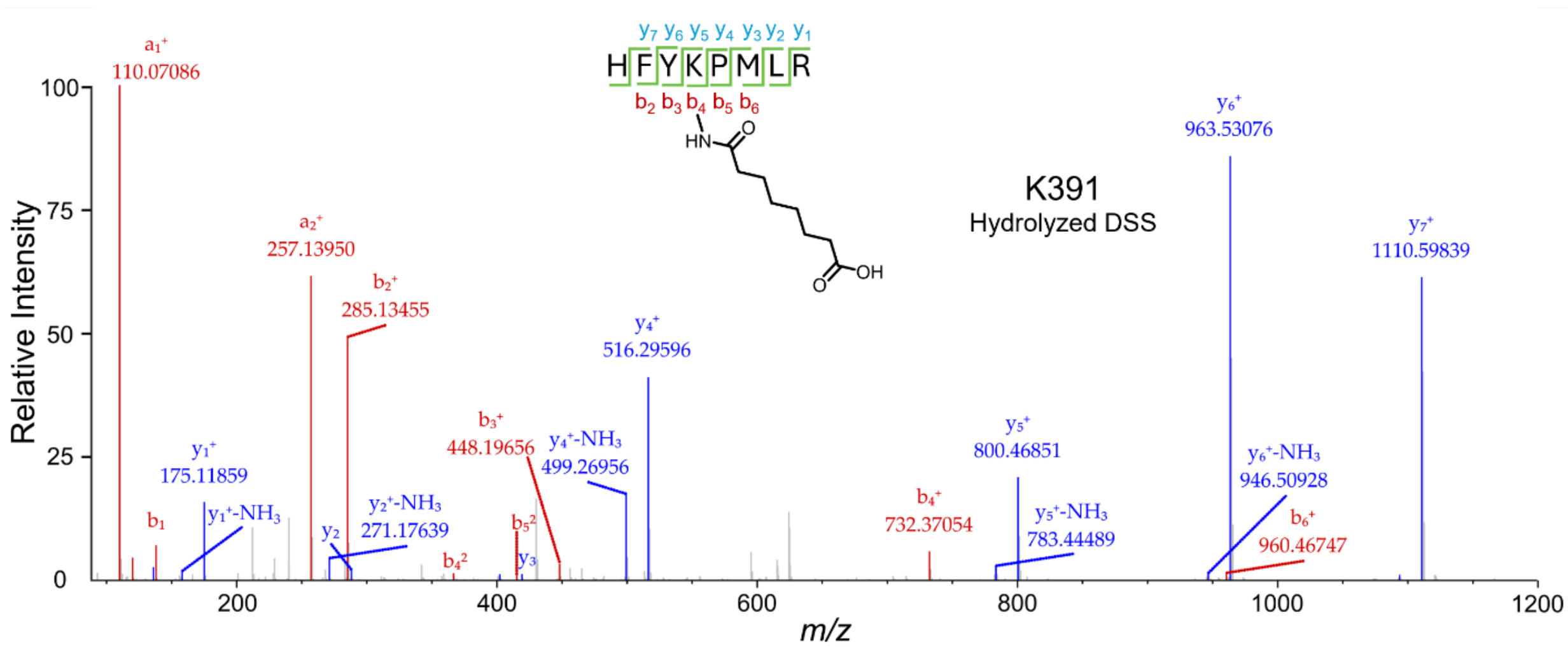

Figure S20. HCD-MS/MS spectrum of hydrolyzed DSS modified peptide at K391 in lipid treated DGAT1.

S11. DGAT1-POPA (PA 16:0_18:1) crosslinking result

The DGAT1 solution was incubated with POPA at a molar ratio of 1:20 (DGAT1: POPA) for 30 min at room temperature to allow lipid binding. DSS was subsequently added at a molar ratio of 1:5 (DGAT1: DSS), and the mixture was incubated for an additional 1 h to facilitate crosslink formation. The resulting samples were analyzed using the bottom-up workflow described above, and peptide-peptide crosslinks were identified using Proteome Discoverer. Because POPA lacks the aziridine functional group required for reaction with the crosslinker, only peptide-peptide crosslinking data could be obtained. Crosslinks are shown only if they passed 1% FDR filtering and were observed in at least 2 of 3 biological replicates. The K391-K224 and K53-K231 peptide-peptide crosslinks were also observed in POPA-treated DGAT1, consistent with the Azi-PA result. These data suggest that the observed conformational change is induced by PA binding rather than by the aziridination modification.

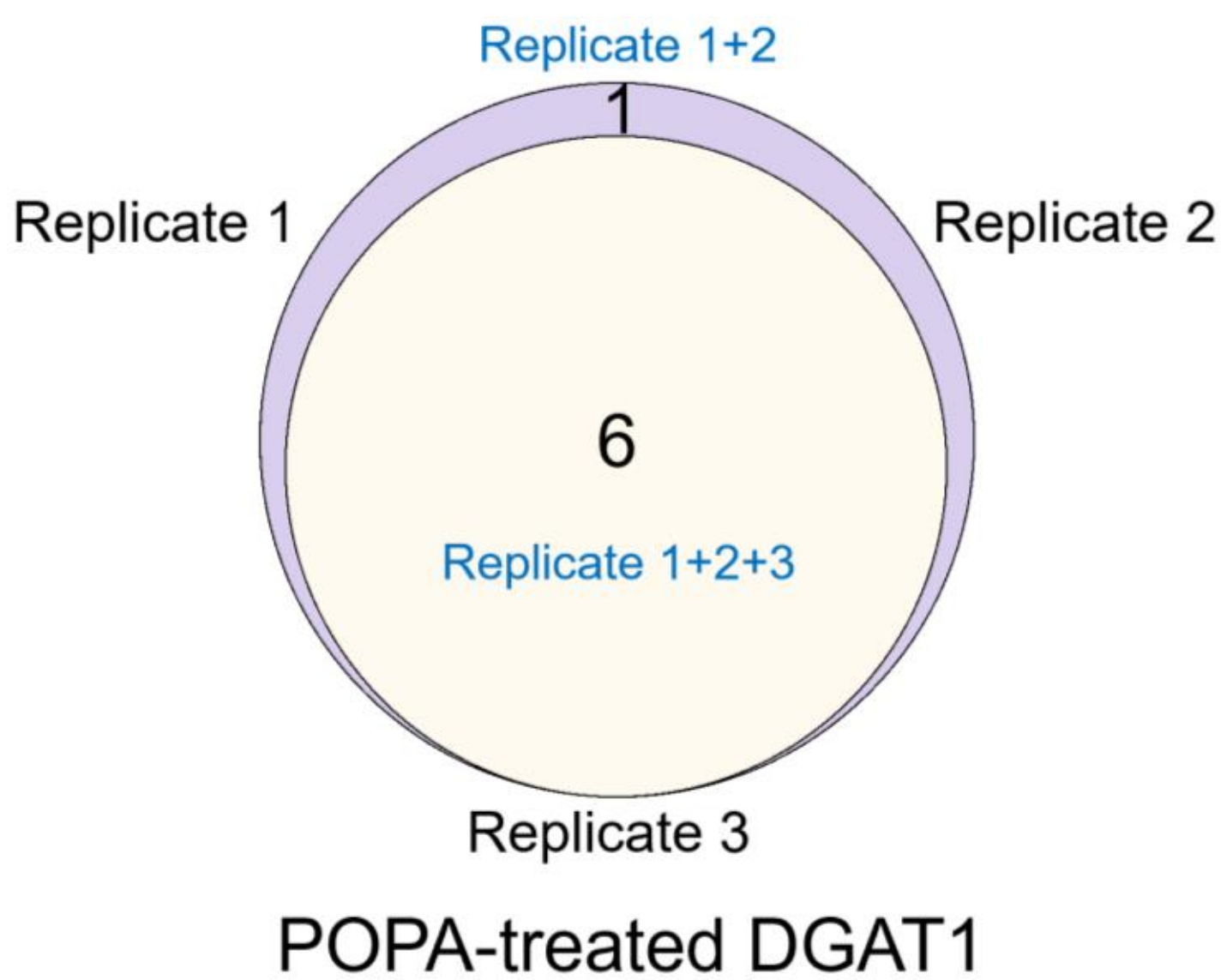

Figure S21. Overlap of crosslinked residue pairs identified across biological triplicates for POPA-treated DGAT1.

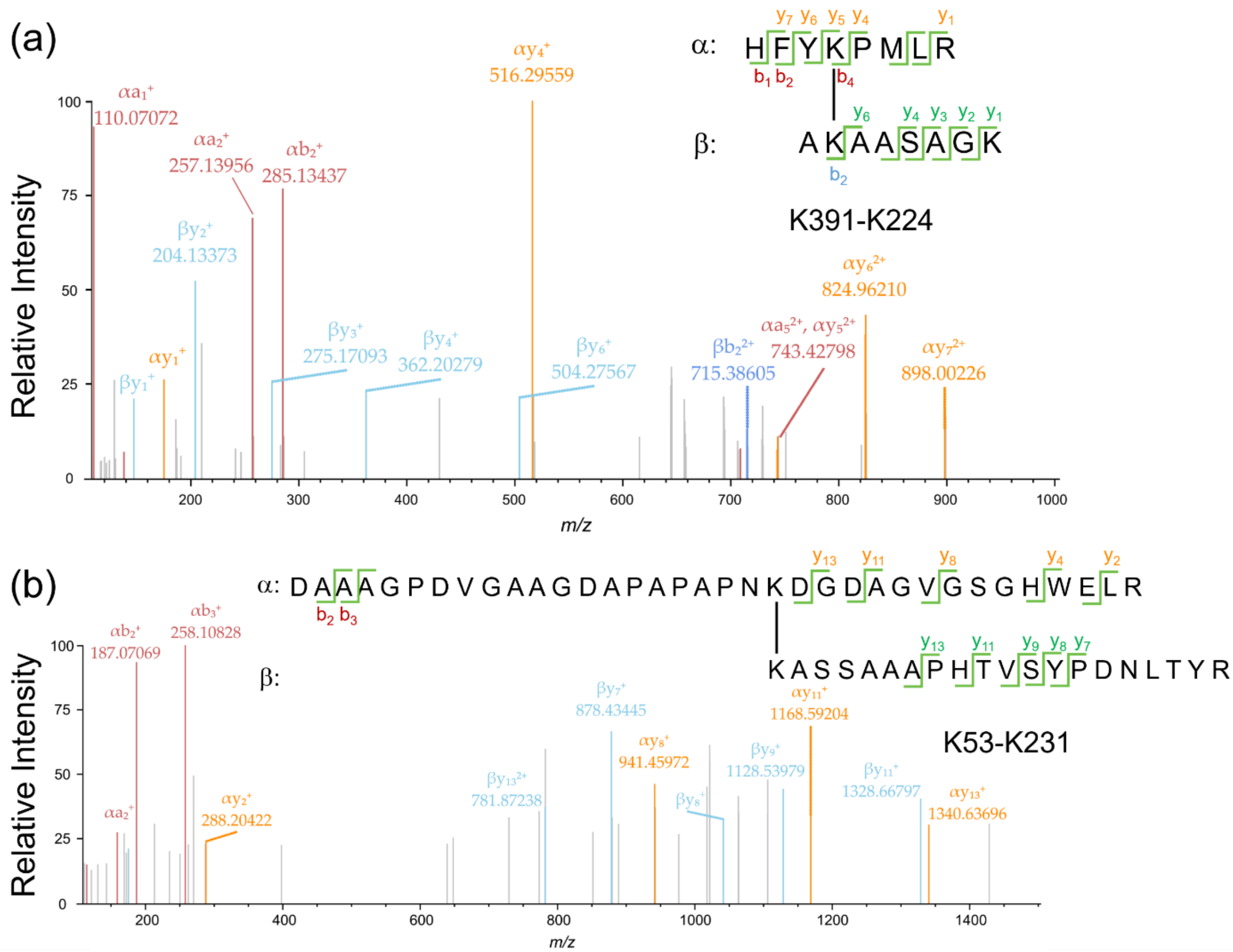

Figure S22. HCD-MS/MS spectra of peptide–peptide crosslinked products in POPA-treated DGAT1 at (a) K391-K224 and (b) K53-231.

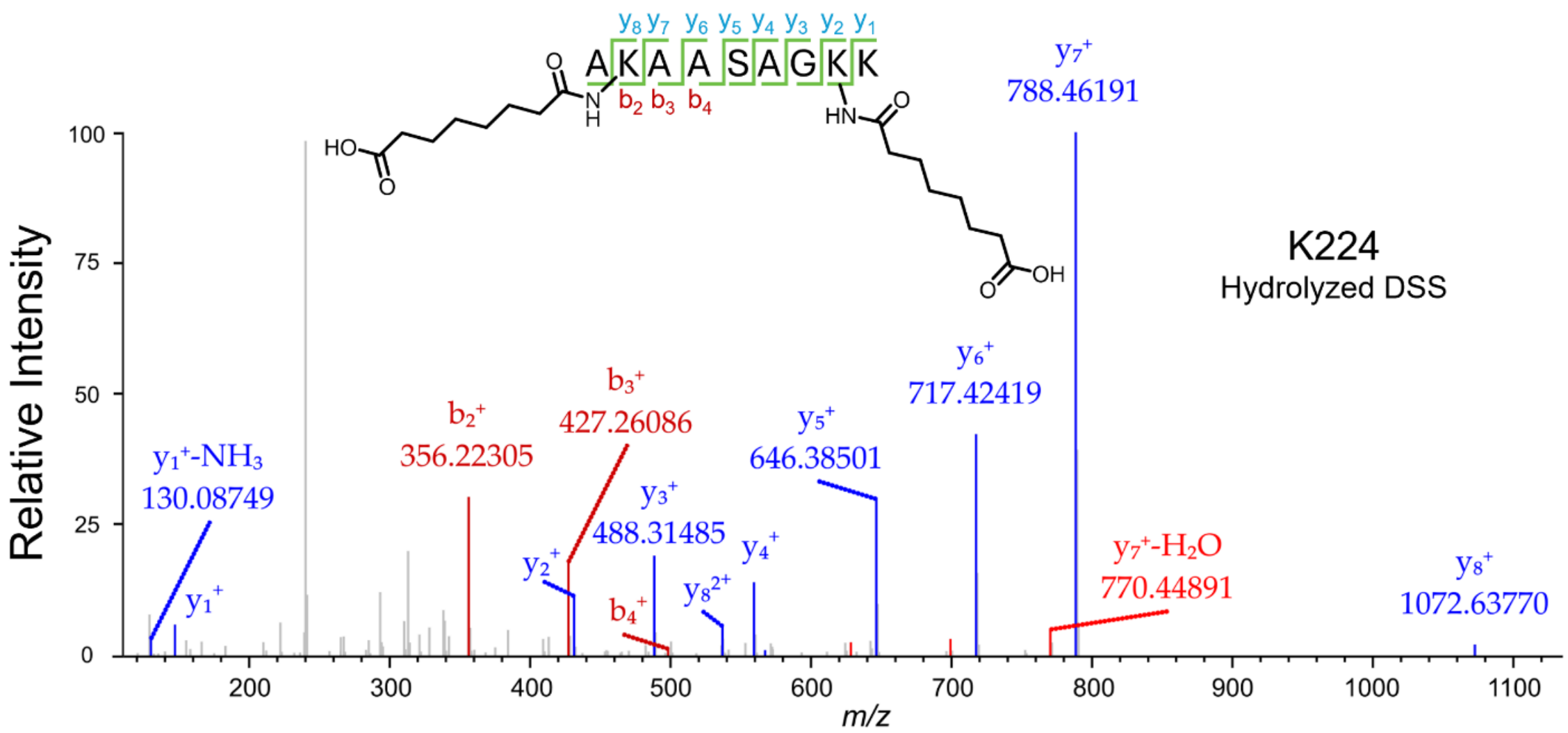

Figure S23. HCD-MS/MS spectrum of hydrolyzed DSS modified peptide at K224 in POPA-treated DGAT1.

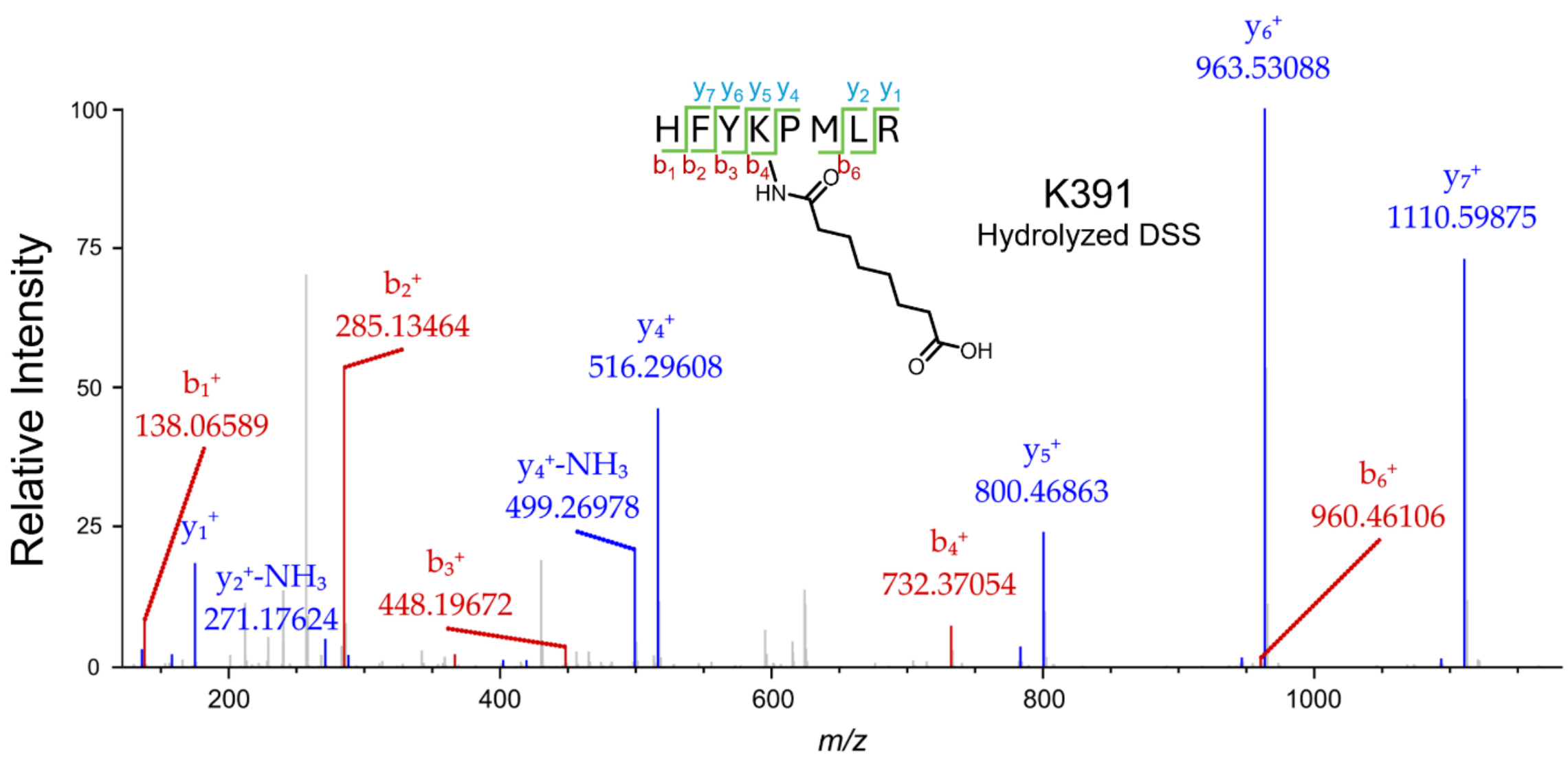

Figure S24. HCD-MS/MS spectrum of hydrolyzed DSS modified peptide at K391 in POPA-treated DGAT1.

S12. Additional MS/MS Spectral Analysis of Crosslinks

The additional MS/MS spectral analysis of crosslinks showed that while application of the more stringent XlinkX threshold removed a number of lower-confidence candidates, a small number of crosslinks with XlinkX scores below 40 were nevertheless supported by high-quality MS/MS evidence. For example, the K391-K400 crosslink identified in both apo DGAT1 and lipid-treated DGAT1 exhibited comparable precursor mass accuracy (−0.10 and 2.73 ppm, respectively) and similar sequence fragment ion coverage, yet received XlinkX scores of 25.0 and 46.35, respectively (Figure S25). This example illustrates that the XlinkX score is an important confidence metric but is most appropriately interpreted together with FDR control and careful spectral evaluation rather than used as the sole criterion for crosslink acceptance, consistent with current recommendations for XL-MS data validation (Iacobucci, C.; Sinz, A. *Anal. Chem.* **2017**, 89, 7832–7835).[5]

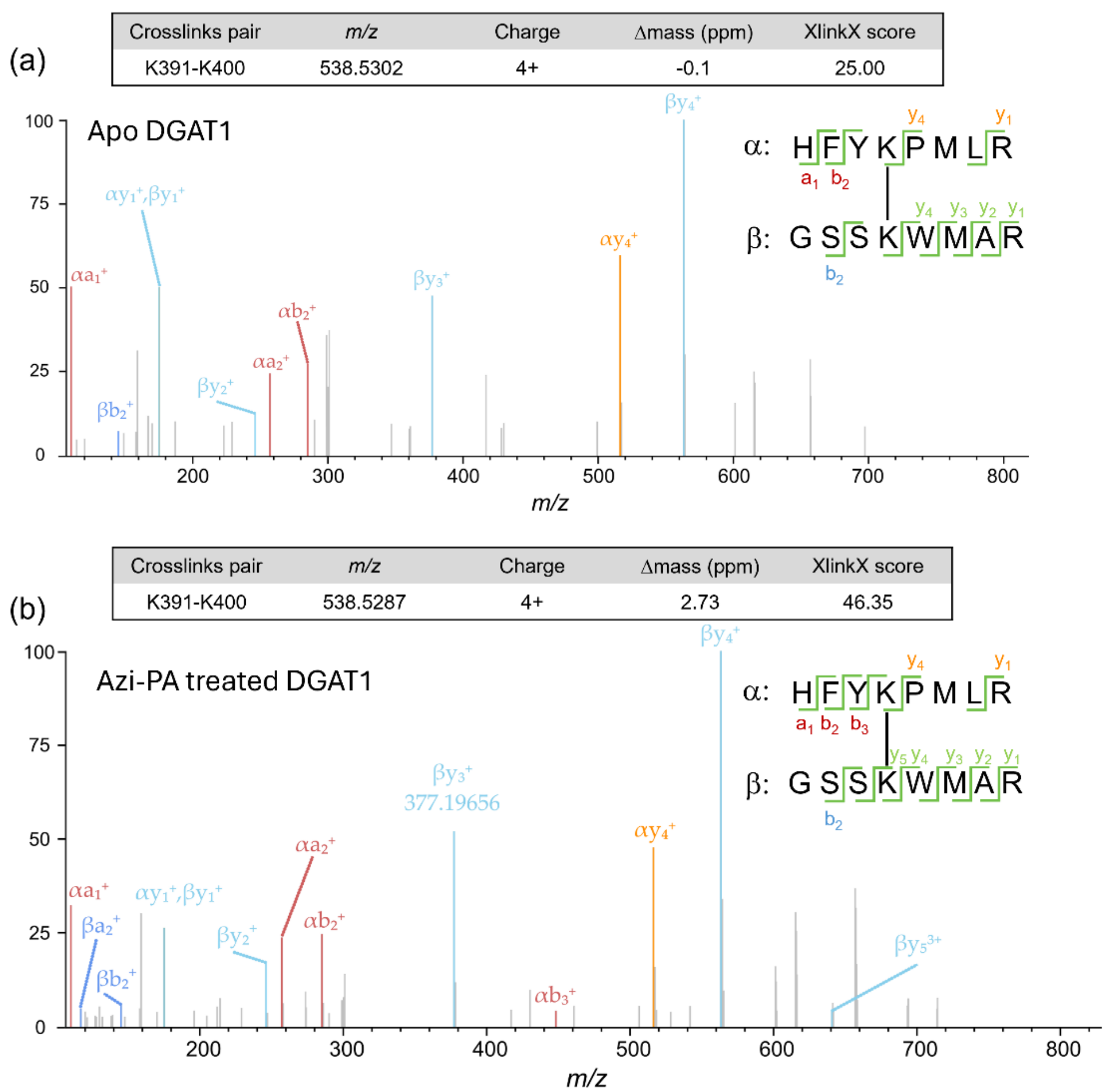

(a)

Crosslinks pair	*m/z*	Charge	Δmass (ppm)	XlinkX score
K391-K400	538.5302	4+	-0.1	25.00

(b)

Crosslinks pair	*m/z*	Charge	Δmass (ppm)	XlinkX score
K391-K400	538.5287	4+	2.73	46.35

Figure S25. HCD-MS/MS spectra of the K391–K400 crosslink identified in (a) apo DGAT1 and (b) Azi-PA-treated DGAT1.

Apo BLG

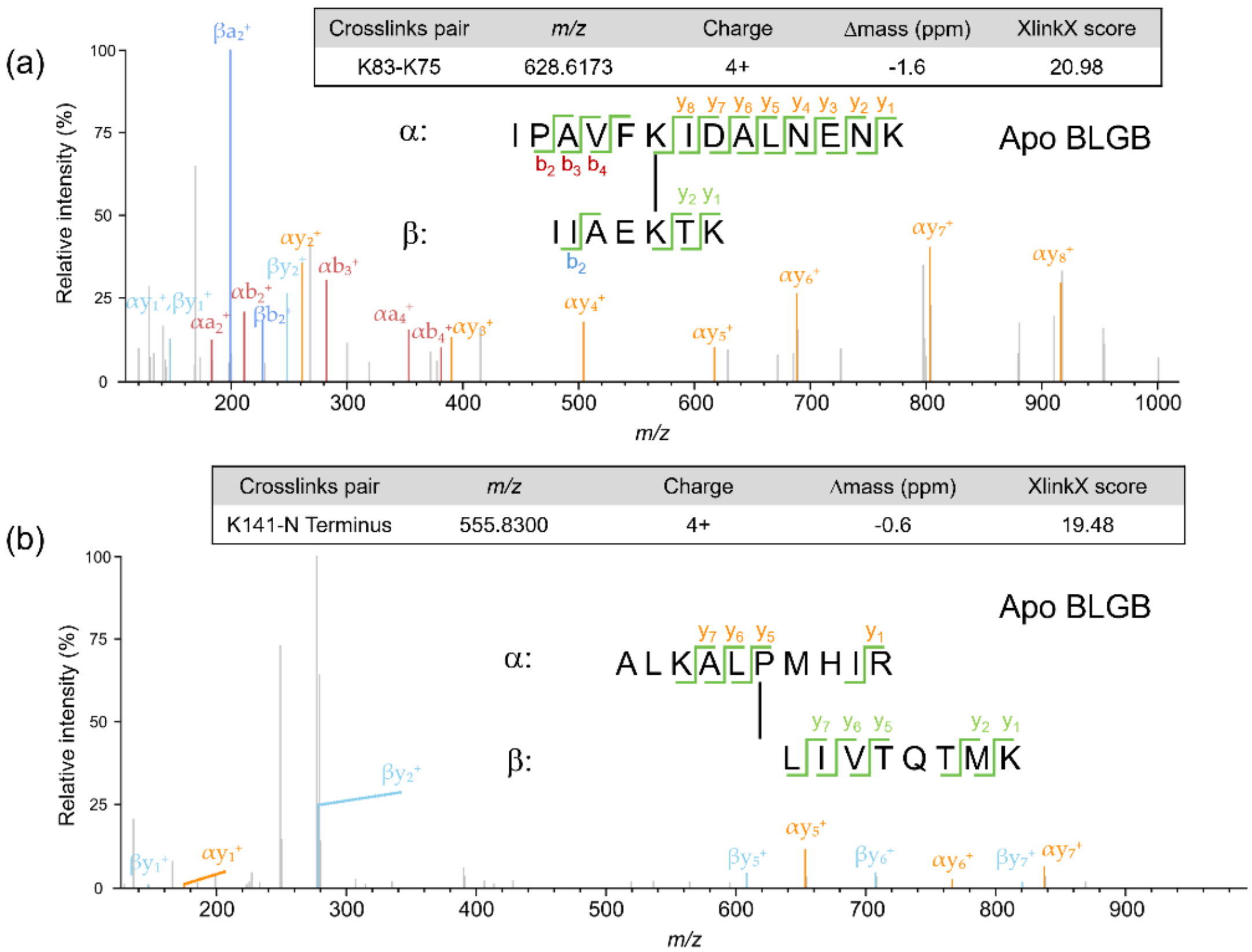

Figure S26. HCD-MS/MS spectra of the (a) K391-K400 (b) K141-N-terminus crosslinks.

Lipid-treated BLG

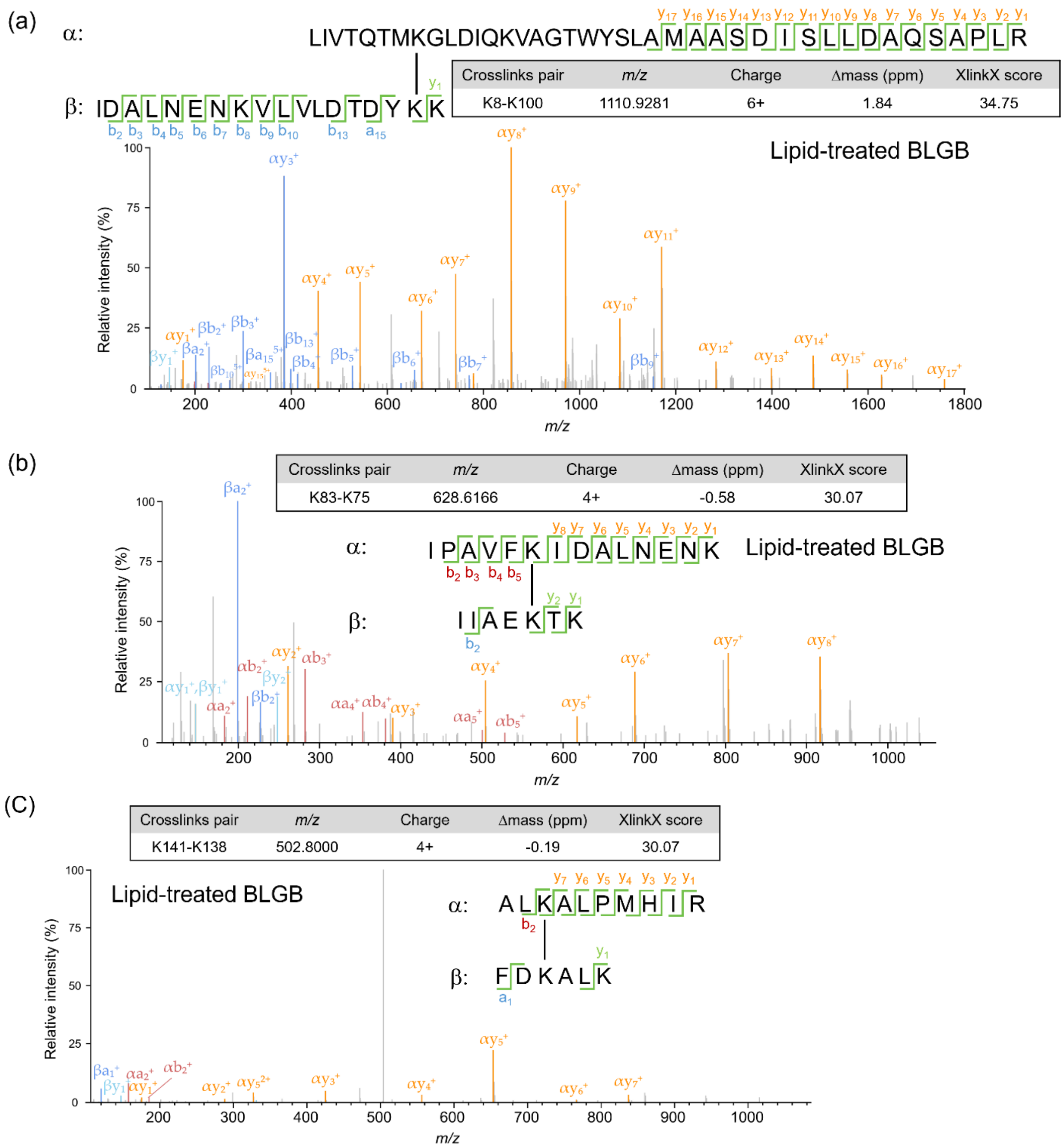

(a)

Crosslinks pair	m/z	Charge	Δmass (ppm)	XlinkX score
K8-K100	1110.9281	6+	1.84	34.75

(b)

Crosslinks pair	m/z	Charge	Δmass (ppm)	XlinkX score
K83-K75	628.6166	4+	-0.58	30.07

(C)

Crosslinks pair	m/z	Charge	Δmass (ppm)	XlinkX score
K141-K138	502.8000	4+	-0.19	30.07

Figure S27. HCD-MS/MS spectra of the (a) K8-K100, (b) K83-K75 and (c) K141-K138 crosslinks.

Apo DGAT1

(a)

Crosslinks pair	*m/z*	Charge	Δmass (ppm)	XlinkX score
K53-K224	822.8032	5+	1.00	32.97

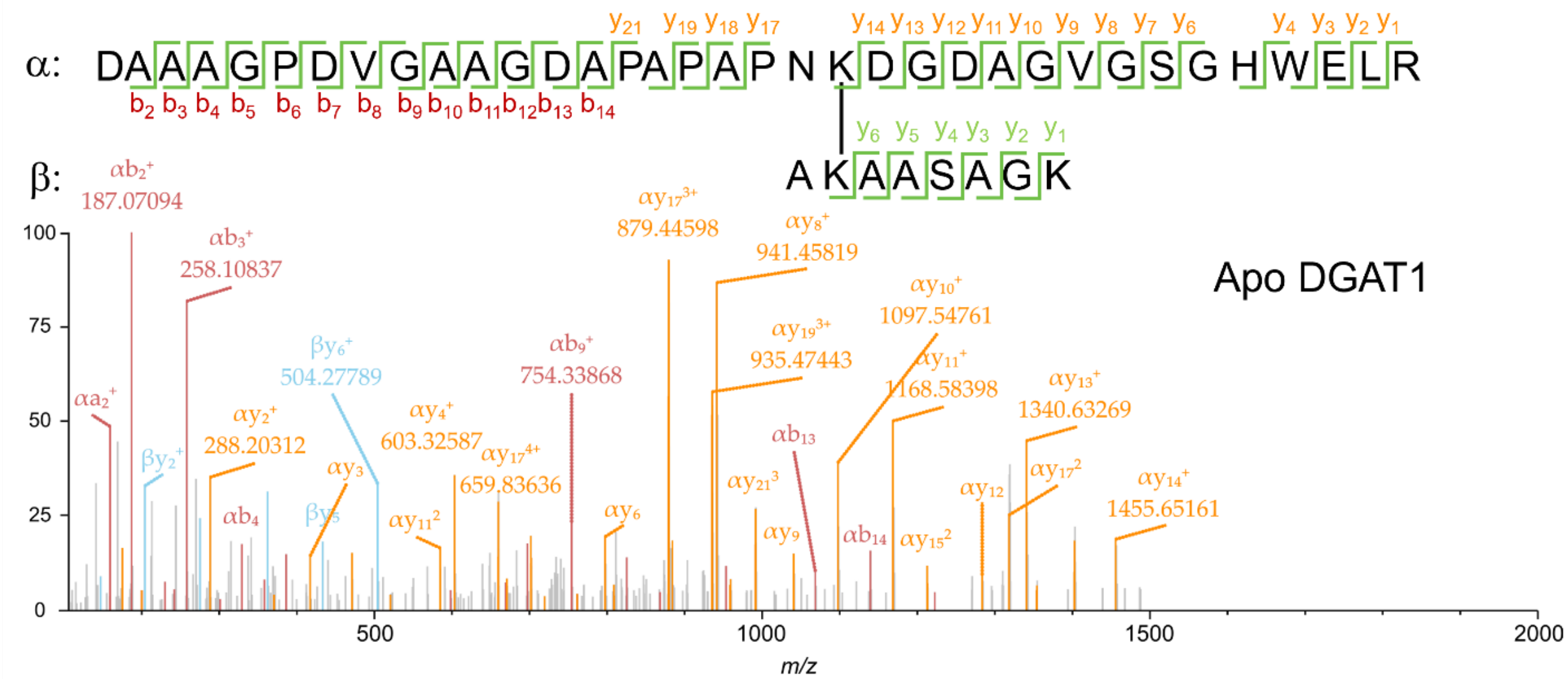

(b)

Crosslinks pair	*m/z*	Charge	Δmass (ppm)	XlinkX score
K53-K391	900.4378	5+	0.75	18.81

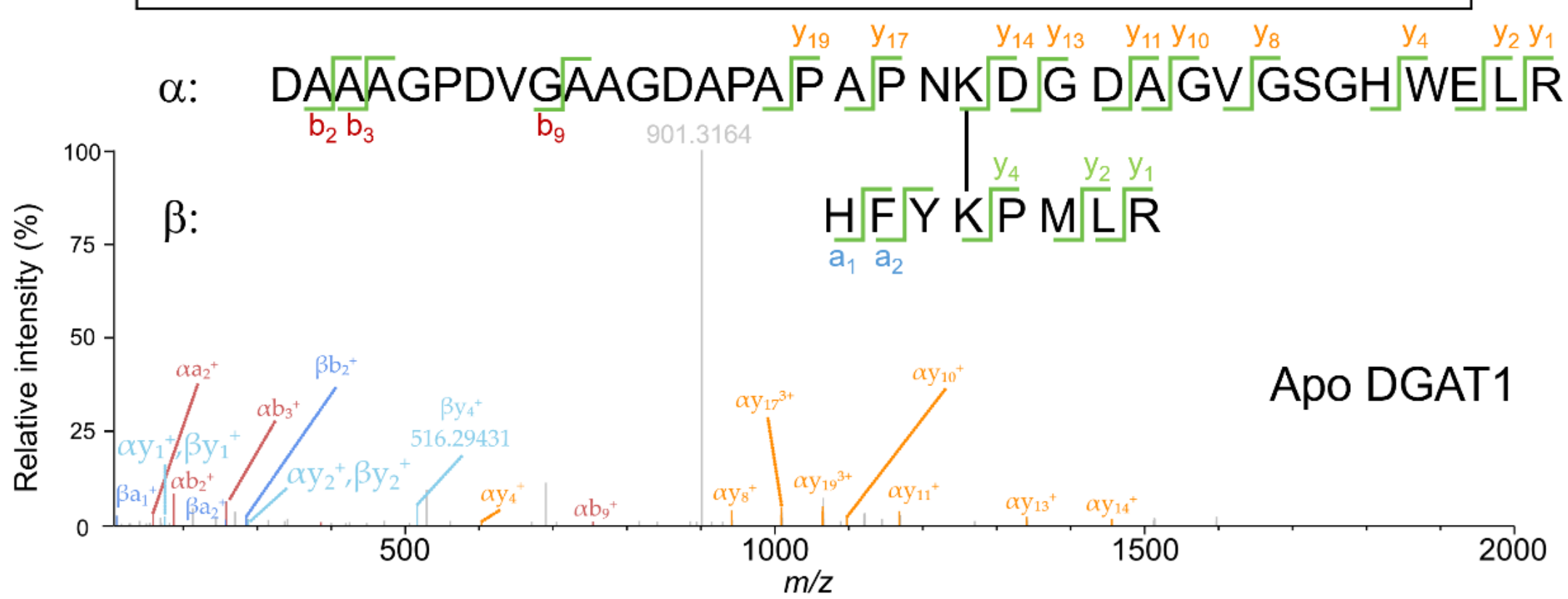

Figure S28. HCD-MS/MS spectra of the (a) K53-K224 and (b) K53-391 crosslinks.

Azi-PA-treated DGAT1

(a)

Crosslinks pair	*m/z*	Charge	Δmass (ppm)	XlinkX score
K53-K391	900.4373	5+	1.30	36.56

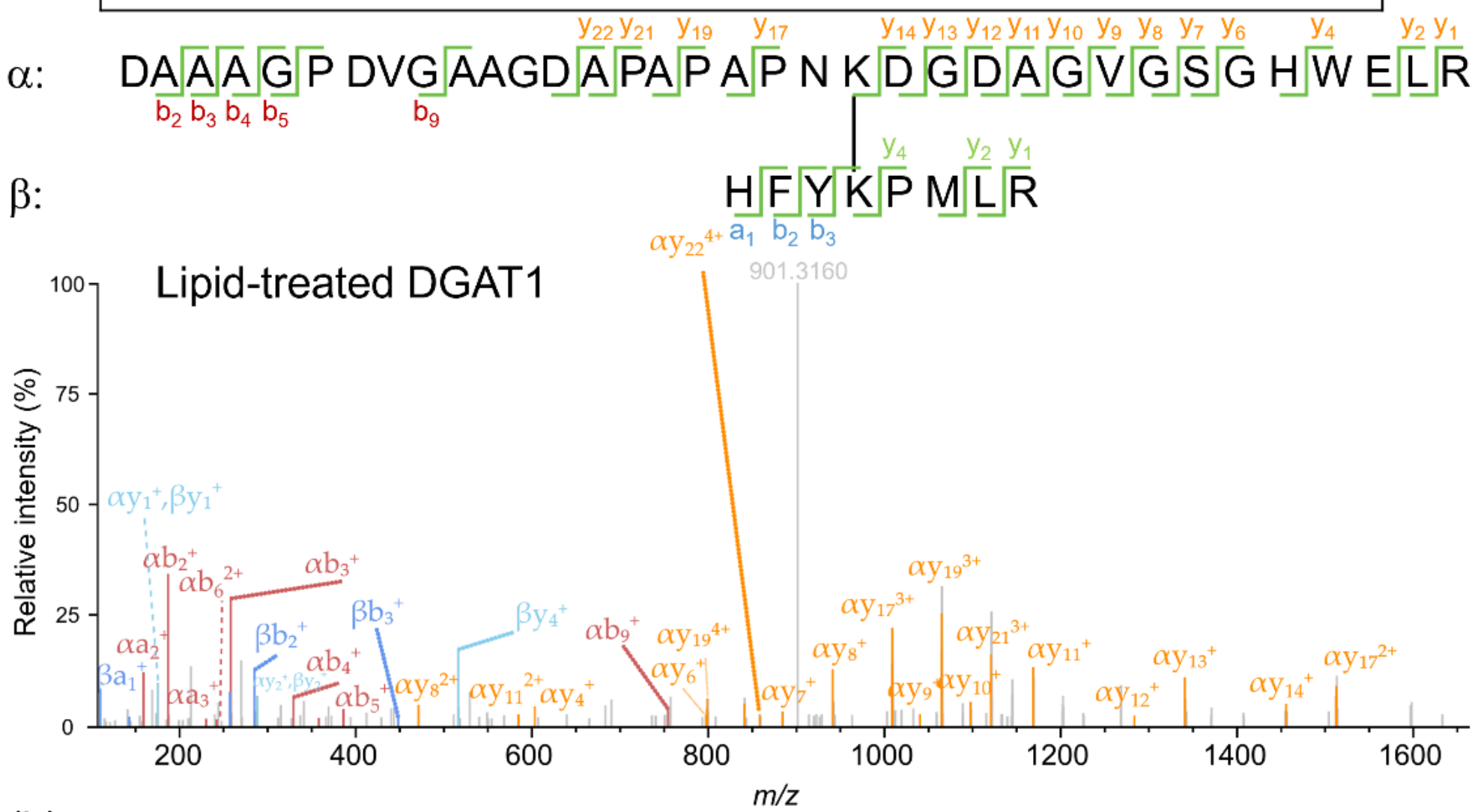

(b)

Crosslinks pair	*m/z*	Charge	Δmass (ppm)	XlinkX score
K391-K224	483.7690	4+	-0.69	36.56

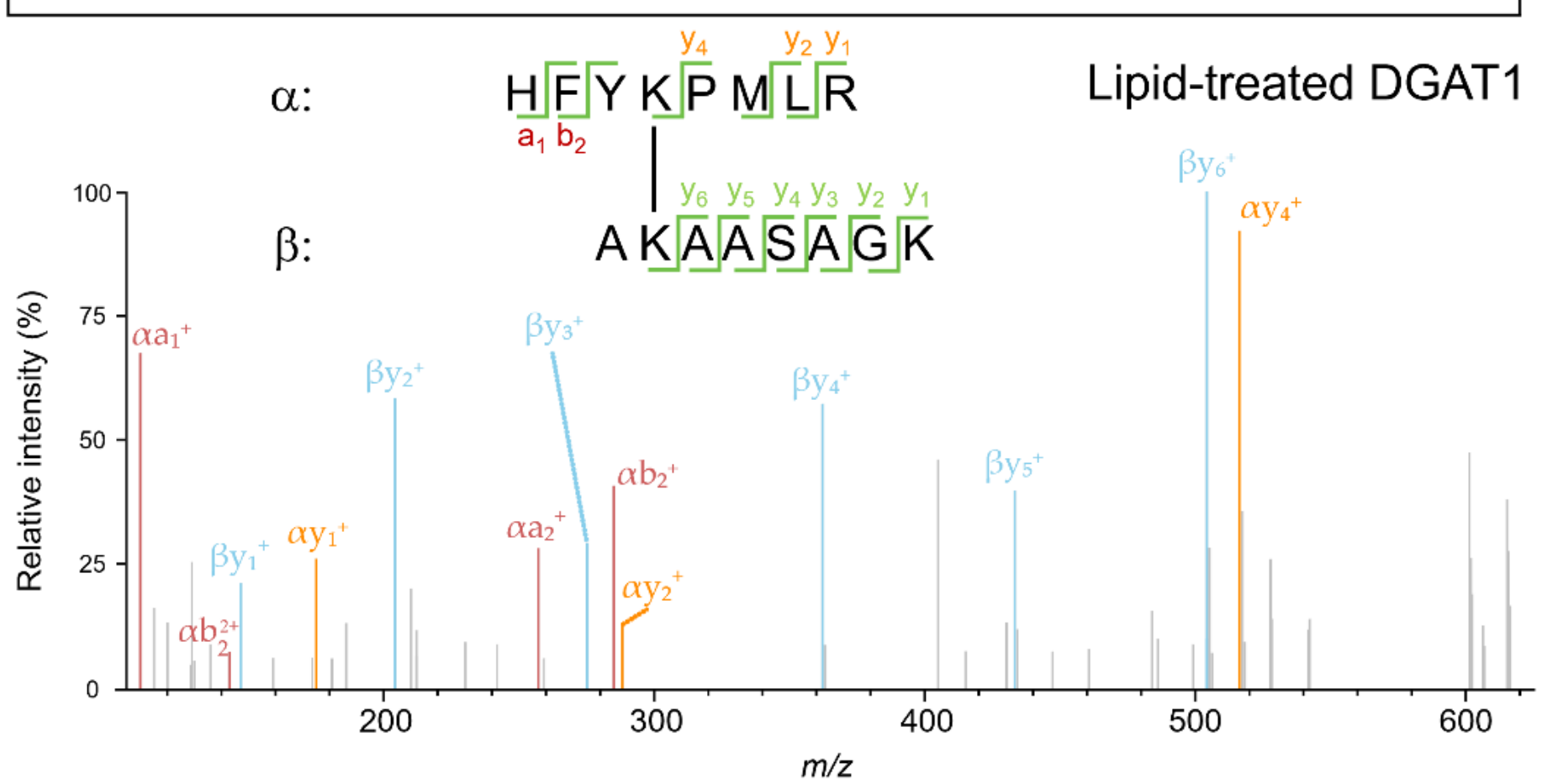

Figure S29. HCD-MS/MS spectra of the (a) K53-K391 and (b) K391-K224 crosslinks.

POPA-treated DGAT1

Crosslinks pair	*m/z*	Charge	Δmass (ppm)	XlinkX score
K53-K224	822.8024	5+	1.97	39.02

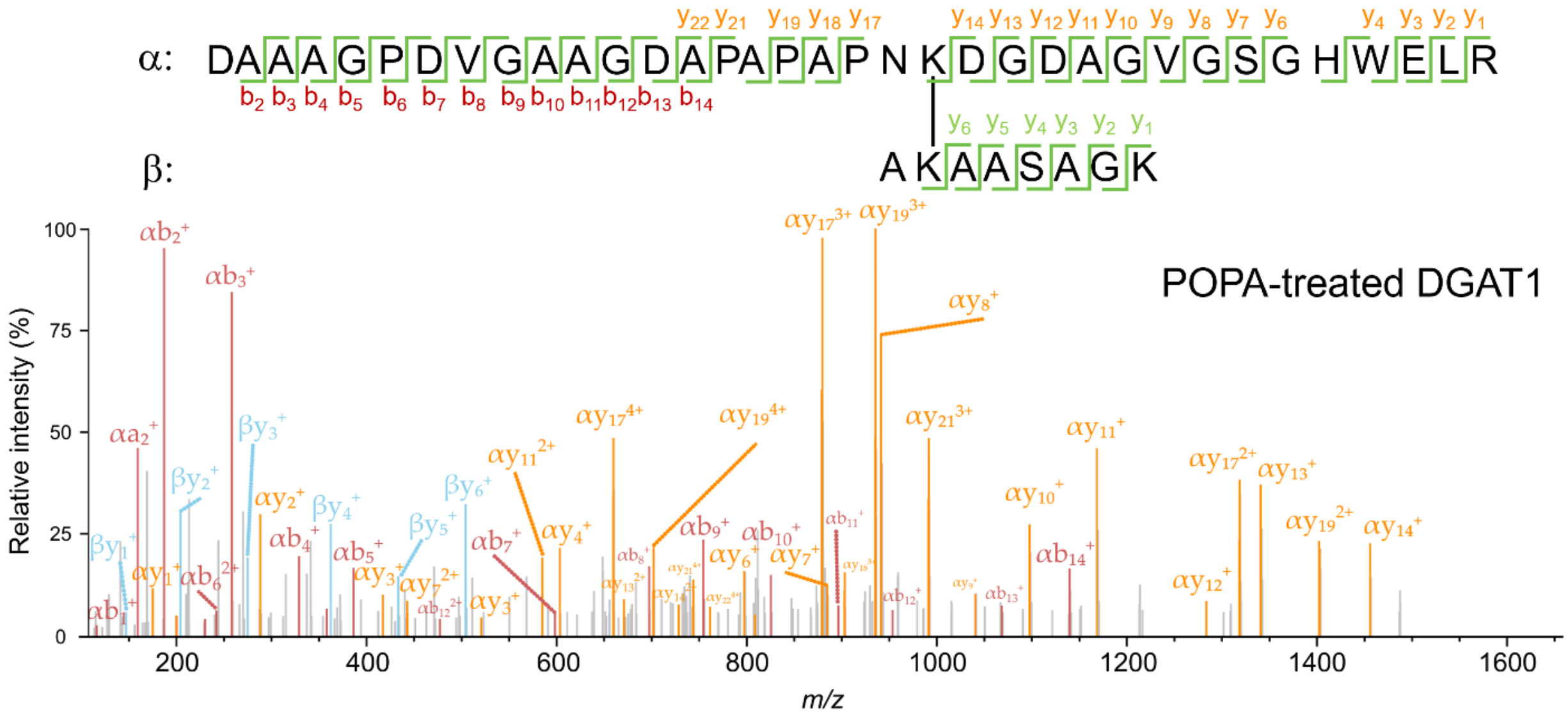

Figure S30. HCD-MS/MS spectrum of the K53-K224 crosslink.

S13. Simulation supplementary data

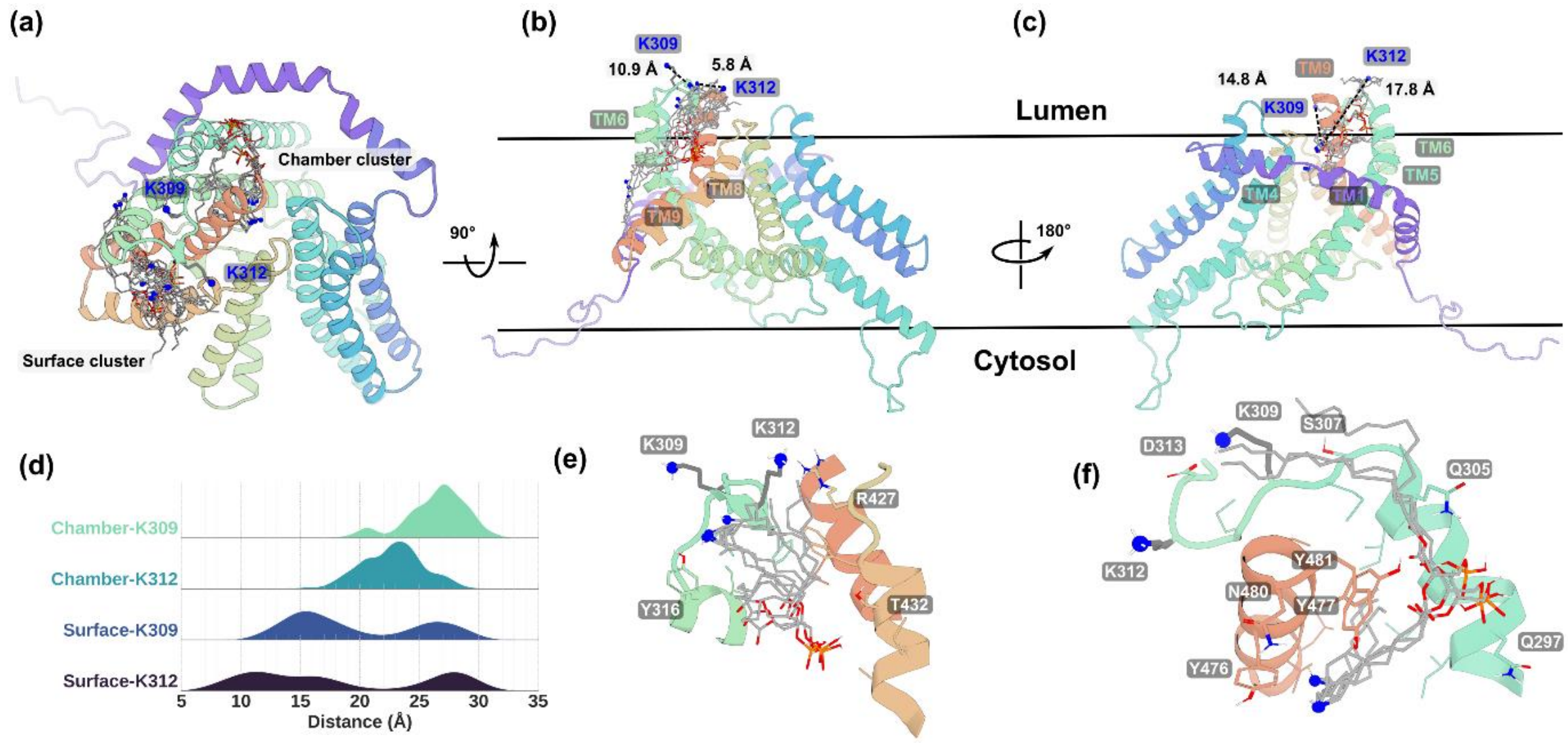

Figure S31. Azi-PA docking and binding modes in DGAT1. (a) Overall view of DGAT1 with docked Azi-PA poses, which are grouped into two binding orientations, designated the surface cluster and chamber cluster. (b) Front view of representative surface-cluster poses. Surrounding transmembrane helices are labeled TM1–TM9 according to their sequence order. (c) Back view of representative chamber-cluster poses. (d) Distance distributions for protein-ligand pairs. The surface cluster shows broader distributions, indicating greater ligand mobility. (e) Residue contact map for surface-cluster poses. (f) Residue contact map for chamber-cluster poses. Polar residues are labeled and shown as sticks, whereas hydrophobic residues are shown as lines.

Table S1. Contact map of docked poses using a 4 Å cutoff to define ligand-protein contacts.

Pose	Cluster	Number of contacts	%hydrophobic	%polar	Hydrophobic residues	Polar residues
1	Chamber	28	71%	29%	P191, V192, L195, V293, I296, M300, V301, I304, F311, L326, I333, W334, F337, L418, V419, P422, L423, V469, L470, V473	Q292, Q297, T303, R322, N330, H474, Y476, Y477
2	Chamber	18	50%	50%	I296, M300, V301, I304, M308, P310, L470, V473, V478	Q297, Q305, S307, K309, D313, Y476, Y477, N480, Y481
3	Surface	12	67%	33%	F311, M314, I319, W429, G433, M435, A436, M471	K312, Y316, R427, T432
4	Surface	17	82%	18%	F311, I319, I320, L324, W429, G433, A436, L440, F443, I462, I463, I467, A468, M471	K312, Y316, T432
5	Surface	23	83%	17%	F311, I319, I320, L323, L324, A327, V328, F426, W429, G433, F448, A455, A456, W458, L459, I462, I463, A468, M471	K312, R427, T432, Y452

References:

(1) Sui, X.; Wang, K.; Gluchowski, N. L.; Elliott, S. D.; Liao, M.; Walther, T. C.; Farese, R. V. Structure and catalytic mechanism of a human triacylglycerol-synthesis enzyme. *Nature* **2020**, *581* (7808), 323–328. DOI: 10.1038/s41586-020-2289-6.
(2) Sui, X.; Wang, K.; Song, K.; Xu, C.; Song, J.; Lee, C.-W.; Liao, M.; Farese, R. V.; Walther, T. C. Mechanism of action for small-molecule inhibitors of triacylglycerol synthesis. *Nat. Commun.* **2023**, *14* (1), 3100. DOI: 10.1038/s41467-023-38934-3.
(3) Sapia, J.; Salo, V. T.; Tasnim, A. T.; Campomanes, P.; Sui, X.; Vanni, S. Substrate-dependent oligomerization modulates DGAT1 activity and subcellular localization. *bioRxiv* **2026**, 2026.2003.2031.715571. DOI: 10.64898/2026.03.31.715571.
(4) Marty, M. T.; Baldwin, A. J.; Marklund, E. G.; Hochberg, G. K. A.; Benesch, J. L. P.; Robinson, C. V. Bayesian Deconvolution of Mass and Ion Mobility Spectra: From Binary Interactions to Polydisperse Ensembles. *Anal. Chem.* **2015**, *87* (8), 4370–4376. DOI: 10.1021/acs.analchem.5b00140.
(5) Iacobucci, C.; Sinz, A. To Be or Not to Be? Five Guidelines to Avoid Misassignments in Cross-Linking/Mass Spectrometry. *Analytical Chemistry* **2017**, *89* (15), 7832–7835. DOI: 10.1021/acs.analchem.7b02316 (accessed 8/2/2026).
(6) Abramson, J.; Adler, J.; Dunger, J.; Evans, R.; Green, T.; Pritzel, A.; Ronneberger, O.; Willmore, L.; Ballard, A. J.; Bambrick, J.; et al. Accurate structure prediction of biomolecular interactions with AlphaFold 3. *Nature* **2024**, *630* (8016), 493–500. DOI: 10.1038/s41586-024-07487-w.
(7) Eswar, N.; Webb, B.; Marti-Renom, M. A.; Madhusudhan, M. S.; Eramian, D.; Shen, M.-y.; Pieper, U.; Sali, A. Comparative Protein Structure Modeling Using Modeller. *Current Protocols in Bioinformatics* **2006**, *15* (1), 5.6.1–5.6.30. DOI: https://doi.org/10.1002/0471250953.bi0506s15 (accessed 2026/04/08).
(8) Vanommeslaeghe, K.; Hatcher, E.; Acharya, C.; Kundu, S.; Zhong, S.; Shim, J.; Darian, E.; Guvench, O.; Lopes, P.; Vorobyov, I.; et al. CHARMM general force field: A force field for drug-like molecules compatible with the CHARMM all-atom additive biological force fields. *J Comput Chem* **2010**, *31* (4), 671–690. DOI: 10.1002/jcc.21367 From NLM.
(9) Jo, S.; Kim, T.; Iyer, V. G.; Im, W. CHARMM-GUI: A web-based graphical user interface for CHARMM. *Journal of Computational Chemistry* **2008**, *29* (11), 1859–1865. DOI: https://doi.org/10.1002/jcc.20945.
(10) Trott, O.; Olson, A. J. AutoDock Vina: improving the speed and accuracy of docking with a new scoring function, efficient optimization, and multithreading. *J Comput Chem* **2010**, *31* (2), 455–461. DOI: 10.1002/jcc.21334 From NLM.
(11) Eberhardt, J.; Santos-Martins, D.; Tillack, A. F.; Forli, S. AutoDock Vina 1.2.0: New Docking Methods, Expanded Force Field, and Python Bindings. *J Chem Inf Model* **2021**, *61* (8), 3891–3898. DOI: 10.1021/acs.jcim.1c00203 From NLM.
(12) Abraham, M. J.; Murtola, T.; Schulz, R.; Páll, S.; Smith, J. C.; Hess, B.; Lindahl, E. GROMACS: High performance molecular simulations through multi-level parallelism from laptops to supercomputers. *SoftwareX* **2015**, *1-2*, 19–25. DOI: https://doi.org/10.1016/j.softx.2015.06.001.
(13) Huang, J.; Rauscher, S.; Nawrocki, G.; Ran, T.; Feig, M.; de Groot, B. L.; Grubmüller, H.; MacKerell, A. D. CHARMM36m: an improved force field for folded and intrinsically disordered proteins. *Nature Methods* **2017**, *14* (1), 71–73. DOI: 10.1038/nmeth.4067.

(14) Jorgensen, W. L.; Chandrasekhar, J.; Madura, J. D.; Impey, R. W.; Klein, M. L. Comparison of simple potential functions for simulating liquid water. *The Journal of Chemical Physics* **1983**, *79* (2), 926–935. DOI: 10.1063/1.445869 (accessed 4/9/2026).
(15) Bussi, G.; Donadio, D.; Parrinello, M. Canonical sampling through velocity rescaling. *The Journal of Chemical Physics* **2007**, *126* (1). DOI: 10.1063/1.2408420 (accessed 5/12/2024).
(16) Parrinello, M.; Rahman, A. Polymorphic transitions in single crystals: A new molecular dynamics method. *Journal of Applied Physics* **1981**, *52* (12), 7182–7190. DOI: 10.1063/1.328693 (accessed 5/12/2024).
(17) Hess, B.; Bekker, H.; Berendsen, H.; Fraaije, J. LINCS: A Linear Constraint Solver for molecular simulations. *Journal of Computational Chemistry* **1998**, *18*. DOI: 10.1002/(SICI)1096-987X(199709)18:123.0.CO;2-H.
(18) Darden, T.; York, D.; Pedersen, L. Particle mesh Ewald: An N·log(N) method for Ewald sums in large systems. *The Journal of Chemical Physics* **1993**, *98* (12), 10089–10092. DOI: 10.1063/1.464397 (accessed 4/9/2026).
(19) Essmann, U.; Perera, L.; Berkowitz, M. L.; Darden, T.; Lee, H.; Pedersen, L. G. A smooth particle mesh Ewald method. *The Journal of Chemical Physics* **1995**, *103* (19), 8577–8593. DOI: 10.1063/1.470117 (accessed 4/9/2026).
(20) Dau, T.; Gupta, K.; Berger, I.; Rappsilber, J. Sequential Digestion with Trypsin and Elastase in Cross-Linking Mass Spectrometry. *Analytical Chemistry* **2019**, *91* (7), 4472–4478. DOI: 10.1021/acs.analchem.8b05222.
(21) Merkley, E. D.; Rysavy, S.; Kahraman, A.; Hafen, R. P.; Daggett, V.; Adkins, J. N. Distance restraints from crosslinking mass spectrometry: Mining a molecular dynamics simulation database to evaluate lysine–lysine distances. *Protein Science* **2014**, *23* (6), 747–759. DOI: https://doi.org/10.1002/pro.2458 (accessed 2026/08/03).
